# Efficacy and safety of laser interstitial thermal therapy versus radiofrequency ablation and stereotactic radiosurgery in the treatment of intractable mesial temporal lobe epilepsy: a systematic review and meta-analysis

**DOI:** 10.1007/s10143-025-03215-8

**Published:** 2025-01-21

**Authors:** Youstina Mohsen, Khalid Sarhan, Ibrahim Saleh Alawadi, Reem Reda Elmahdi, Yasmeena Abdelall Kozaa, Menna A. Gomaa, Ibrahim Serag, Mostafa Shahein

**Affiliations:** 1 Mansoura Manchester Program for Medical Education, Faculty of Medicine, Mansoura University, Mansoura, Egypt; 2 Faculty of Medicine, Mansoura University, Mansoura, Egypt; 3 Department of neurosurgery, Faculty of Medicine, Mansoura University, Mansoura, Egypt

**Keywords:** Mesial temporal lobe epilepsy, Intractable epilepsy, Laser interstitial thermal therapy, Radiofrequency ablation, Stereotactic radiosurgery

## Abstract

Epilepsy is a common neurological disease that is treated with medications; however, patients with drug-resistant epilepsy, commonly intractable temporal lobe epilepsy, tend to have better control with surgical treatment. While the mainstay of surgical treatment is anterior temporal lobectomy, it carries risk of potential adverse effects hence minimally invasive techniques are now being used as an alternative to open surgery. This systematic review and meta-analysis compare the efficacy and safety of three of the most used techniques: laser interstitial thermal therapy (LITT), radiofrequency ablation (RFA) and stereotactic radiosurgery (SRS). Multiple databases were searched and PRISMA guidelines were followed to make an indirect meta-analysis using the currently available studies for the three techniques in terms of seizure freedom, risk of major complications and reoperations. Further subgroup analyses were carried out for LITT in terms of follow up periods and seizure freedom in patients with or without hippocampal sclerosis. A total of 42 papers were included in this study with a total of 1675 patients (1303 treated by LITT, 188 treated by RFA and 184 treated by SRS). LITT had the highest rate of seizure freedom (55.0%, CI 51.5 − 58.5%, *P* = 0.148), and the lowest rates of major complications (2.3%, CI 1.2 − 3.5%, *P* = 0.070), and reoperations (14.3%, CI 10.4 − 18.3%, *P* = 0.042) compared to RFA and SRS which had seizure freedom of 46.3% (CI 18.8 − 73.7%, *P* = 0.000) and 53.8% (CI 44.4 − 63.3%; I^2 = 40.4%, *P* = 0.098), major complication rate of 3.9% (CI 0.7 − 7.0%; I^2 = 0%, *P* = 0.458) and 14.3% (CI 3.1 − 25.5%, *P* = 0.000) and reoperation rate of 28.6% (CI -4.3 − 61.5%, *P* = 0.000) and 15.4% (CI 6.1 − 24.8%; I^2 = 0%, *P* = 0.392) respectively. In conclusion, LITT showed the highest rate of seizure freedom and the lowest rate of major complications and reoperations compared to RFA and SRS in single-arm studies of intractable mesial temporal lobe epilepsy. However, the lack of direct comparisons between treatment modalities limits definitive conclusions. Despite this, LITT appears to offer a favorable safety profile, warranting further prospective, comparative studies to validate these findings.

**Supplementary Information:**

The online version contains supplementary material available at 10.1007/s10143-025-03215-8.

## Introduction

Epilepsy is a prevalent neurological disorder which impacts around 1% of the world’s population. Approximately one-third of these patients have medically refractory epilepsy [1, 2]. Temporal lobe epilepsy is the most common type of focal epilepsy that is medically refractory. Approximately 70% of these cases are mesial temporal lobe epilepsy (mTLE) that are mostly associated with hippocampal sclerosis (HS) [3]. Surgical intervention is a highly effective treatment for mTLE. Surgical approaches for temporal lobectomy have several technical variations including standard anterior temporal lobectomy (ATL), anteromedial temporal lobectomy and selective amygdalohippocampectomy [4–6]. Although standard ATL is the most performed surgical procedure, it can be associated with potential adverse effects including cognitive dysfunction, visual field defects (VFDs), intracranial hemorrhage (ICH), and unintended neurological damage [7]. Minimally invasive therapies including laser interstitial thermal therapy (LITT), Radiofrequency Ablation (RFA) and Stereotactic radiosurgery (SRS) represent promising alternatives to conventional surgery [8–10].

LITT employs thermal energy to induce cell death by damaging DNA and causing protein denaturation [11] aiming to achieve either complete ablation or functional disconnection as a primary treatment goal [12].

LITT provides advantages of being minimally invasive and utilizing real-time magnetic resonance imaging (MRI) for accurate targeting of treatment area [13]. Furthermore MR-guided LITT (MRgLITT) offers shorter recovery period and shorter hospital stays compared to conventional surgery [14]. RFA employs radiofrequency energy to create focal lesions in brain tissue leading to disruption of epileptic circuits [15] while SRS uses stereotactic guidance to transmit focused high dose radiation to particular areas of the brain [16].

### Conflict

ing results were found when comparing the effectiveness and safety of either of the techniques to open surgery, hence it remains a subject of ongoing debate [17–19]. While all three techniques aim to achieve seizure freedom and preserve cognitive function, their comparative effectiveness in terms of long-term seizure control, complication rates, and rates of reoperation remains unclear. Therefore, this systematic review and meta-analysis aims to compare between those three minimal invasive techniques in terms of seizure freedom, major postoperative complications and the rates of reoperation.

## Materials & methods

### Protocol

The present systematic review and meta-analysis was conducted according to PRISMA (Preferred Reporting Items for Systematic Reviews and Meta-Analysis) guidelines and recommendations [20]. Each author worked individually to retrieve the study, filter for eligibility, extract data, and evaluate its quality.

### Search strategy

The PICO model (i.e., population, intervention, comparison, outcome) was adopted to determine the parameters of a search strategy, and the following question was formulated: (population) how do patients with therapy-refractory mTLE (intervention) treated with MRgLITT (comparison) differ from patients who were treated with alternative thermal ablation RFA or SRS (outcome) in terms of seizure freedom, major postoperative complications and rate of reoperations? A systematic search of several databases from each database’s inception to July 2024 in the English language was conducted. The following databases were searched: PubMed, Scopus, Web of Science, and the Cochrane Library and there were no restrictions on publication dates or locations. Controlled vocabulary supplemented with keywords were used in searching. The detailed search strategy containing the used keywords is available in ***Online Resource 1***. The compiled reference lists were then reviewed for potential relevance and the bibliographies of included studies were also searched for missed articles.

### Eligibility criteria

Eligible studies for inclusion in this meta-analysis were required to meet the following criteria: (1) All randomized or non-randomized controlled trials, prospective or retrospective cohort studies and other observational studies, (2) included studies could include either adults or pediatric patients or both, (3) A diagnosis of mTLE was made based on seizure clinical findings, invasive or surface electroencephalogram (EEG) recordings, or morphological findings from MRI, (4) The surgical technique was explicitly described as either MRgLITT, RFA or SRS or any of their corresponding names, (5) The primary seizure outcome was the Engel classification [21], along with other clear outcomes like complications or reoperations. Studies were excluded if one of the following criteria was applied: (1) Non-human studies, case reports, conference abstracts, reviews, editorials, and commentaries, (2) non-English, (3) Those that established a diagnosis of generalized/focal epilepsy other than mTLE, (4) if any of the techniques is used as an adjuvant to previous surgery rather than being an alternative, and (5) studies with sample size less than seven patients were excluded.

### Outcome definition

The main outcome of interest for this study was complete seizure freedom indicated by Engel Classification class I (Engel I) [21]. Since most of the studies supplied patient-level data, we were able to determine the kind of lesion and the seizure frequency outcomes. The rates of postoperative major complications and reoperations were secondary outcomes. Major complications were identified according to the SIR (Society of Interventional Radiology) guidelines as one that “…leads to substantial morbidity and disability […] that increases the level of care, or results in hospital admission or substantially lengthens the hospital stay…” [22]. Since there was no standardized severity assessment among the included studies, complications with permanent VFDs (as homonymous hemianopias and quadrantanopias), infections, or ICH were considered as major complications.

### Study selection

After utilizing Endnote X9 to eliminate duplicate studies, title and abstract screening was performed by two identified independent reviewers then studies were subsequently reviewed according to the defined inclusion and exclusion criteria mentioned above and any differences were resolved by consensus-building with a third reviewer. The following data was extracted and reported by two independent authors: the last name of the first author, the year and where the study was conducted; the sample size; the demographics of the patients (age and sex); the length of time the patients had epilepsy prior to surgery; the length of follow-up period; the type of pathologic condition (HS and non-HS); the left side involvement; the mean ablation volume; major complications and reoperations. Data was extracted from the text, tables, and figures. Variables were calculated using the data that was provided when information was not easily accessible. A third independent reviewer double-checked the data to make sure it was accurate, consistent, and comprehensive.

### Quality assessment and publication bias

The MINORS criteria [23] was used to analyse the risk of bias for non-randomized studies. MINORS criteria is based on eight questions for non-comparative studies (maximum score is 16) and twelve questions for comparative studies (maximum score is 24). For non-comparative studies, score < 9, 9–14, and 15–16, were considered as poor, moderate and good quality, respectively while for comparative studies, < 14, 15–22, and 23–24 respectively, were considered the cut off points. For case series, JBI critical appraisal tool [24] was utilized and for randomized controlled trials (RCTs) included in our study, the ROB-2 tool developed by Cochrane was used to assess their quality [25]. Publication bias was virtually evaluated using a funnel plot with ten or more included papers. To measure any additional bias that was noticed, the Egger test [26] and Begg-Mazumdar test [27] were utilised to assess any further bias seen.

### Statistical analysis

The pooled proportion rate and their respective 95% confidence interval (CI) were computed for dichotomous variables (Engel I outcome, major complications, and reoperation). Any result presented as the median and interquartile range (IQR) was converted to mean and standard deviation (SD) using the Wan et al. [28] method. Because the heterogeneity between studies was predicted, we employed a random-effects model that considers the possible clinical diversity and methodologic variation between studies, because it assumes unequal variance between studies and distributes statistical weighting more conservatively. Heterogeneity was measured using the Higgins I² test and a P-value of < 0.1 as being significant, with 0–40% being regarded as minor, 30–60% as moderate, 50–90% as substantial, and 75–100% as considerable heterogeneity [29]. A comparison between Engel I outcome for the HS and HS negative cohort was carried out and expressed as risk ratio (RR) and 95% CI. In terms of age, epilepsy duration, ablation volume, left side involvement and HS association, a meta-regression analysis was conducted on the seizure freedom rate for the entire cohort. We used Review Manager (V.5.3) and Open Meta-Analyst software (V 10.10) for our statistical analysis. To ensure robustness of our results we underwent sensitivity analysis excluding each study at a time.

## Results

### Study selection and summary of the included studies

**Fig. 1:**
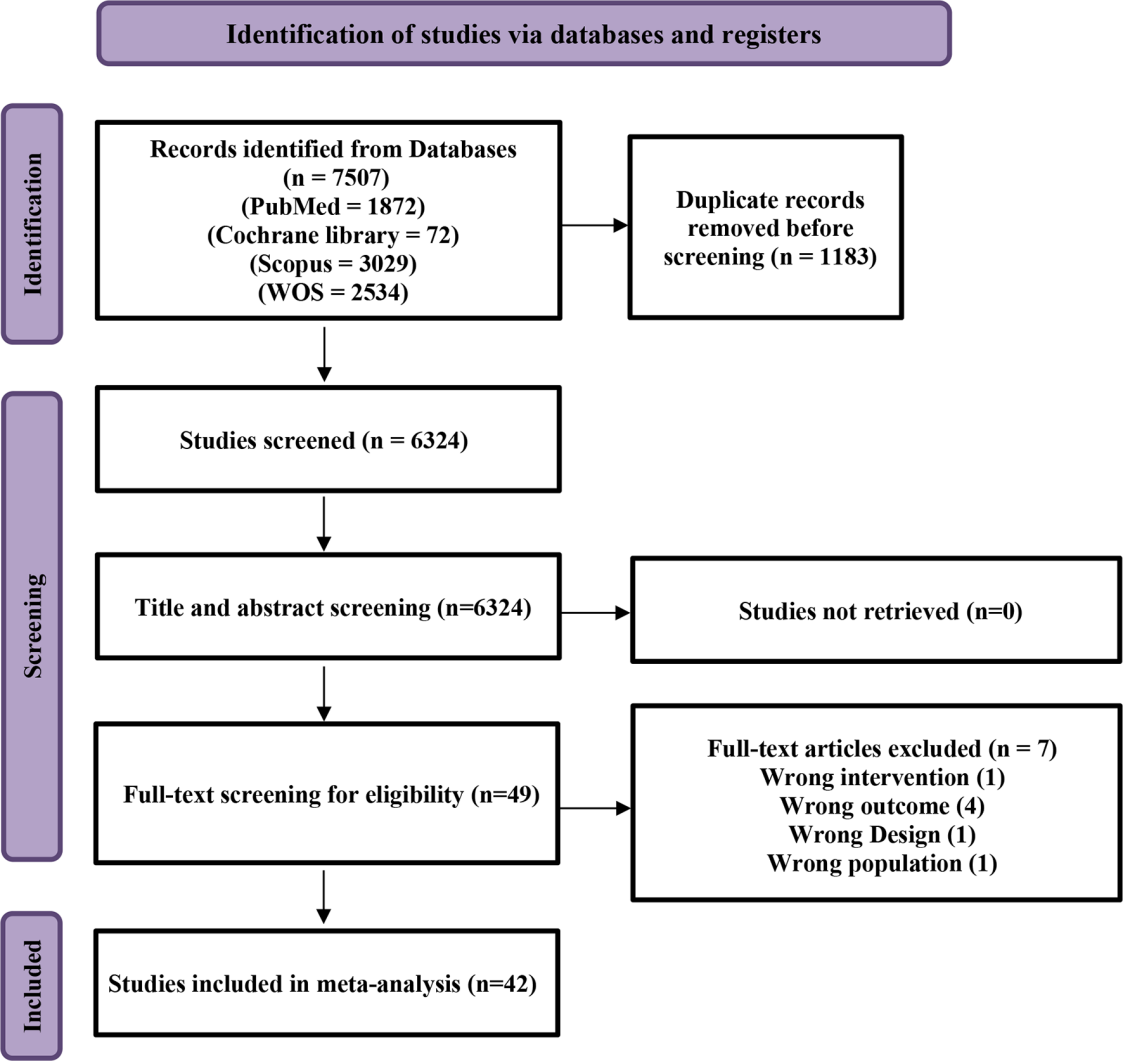
PRISMA flow diagram of the included studies

Using our search strategy, a total of 7507 papers were identified, which came down to 6324 after removal of duplicates. According to the inclusion and exclusion criteria, these papers were screened by title and abstract yielding 49 papers for full text screening. After full text screening, seven papers were excluded yielding a total of 42 papers; 25 for MRgLITT, eight for RFA, and nine for SRS Fig. 1. The study designs included were seven case series, six prospective and 23 retrospective cohorts, three clinical trials and three randomized controlled trials. A detailed summary of the included studies is available in ***Online Resource 2 and 3***.

### Patient characteristics

Twenty-five studies were included for MRgLITT technique with a total of 1303 patients, 48.1% of them were males and 50.6% patients were reported to have left side involvement or had the ablation done on the left side. Their mean age was 38.7 years, mean epilepsy duration before surgery was 23.6 years, and mean total ablated volume was 4.28 cm3. Eight studies were included for RFA technique with a total of 188 patients, 52.7% of them were males and 62.6% patients were reported to have left side involvement or had the ablation done on the left side. Their mean age was 33.4 years, and mean epilepsy duration before surgery was 18.2 years. Nine studies were included for SRS technique with a total of 184 patients, 43.8% of them were males and 49.1% patients were reported to have left side involvement or had the ablation done on the left side. Their mean age was 32.0 years, mean epilepsy duration before surgery was 21.0 years, and mean total ablated volume was 7.46 cm3. For all studies, mean follow up periods were referred to as less than or equal 12 months or more than 12 months. A summary of the baseline characteristics is demonstrated in Table 1 and Table 2.

**Table 1:** Baseline characteristics of included studies for laser interstitial thermal therapy

Study ID	Study design	Sample size	Male (*n* %)	Mean age (years)	Mean follow up (months)		Mean epilepsy duration (years)	HS: non- HS	Left side involved (*n* %)	Mean ablation volume (cm^3^)
*Cajigas et al.*,*2019* [38]	Case series	26	14 (53.8%)	43.8		42.9	28.7	19: 7	14 (53.9%)	5.4
*Chen et al.*,*2023* [45]	Prospective cohort	7	5 (71.4%)	44.7		23.7	29.5	3: 4	5 (71.4%)	4.15
*Donos et al.*,*2018* [37]	Case series	43	23 (53.49%)	39.8		20.3	22.6	34: 9	N/A	N/A
*Drane et al.*,*2015* [46]	Prospective cohort	19	N/A	38.2		6	N/A	10: 9	N/A	N/A
*Drane et al.*,*2021* [47]	Retrospective cohort	40	N/A	40		12	23.35	24: 16	19 (47.5%)	N/A
*Greenway et al.*,*2017* [39]	Case series	15	8 (53.34%)	44.6		26	N/A	11: 4	9 (60%)	N/A
*Grewal et al.*,*2018* [48]	Retrospective cohort	23	10 (43%)	43.9		37.5	N/A	18: 5	15 (65.2%)	6.888
*Gross et al.*,*2018* [49]	Retrospective cohort	58	25 (43.1%)	40		12	N/A	43: 15	28 (48.3%)	N/A
*Jermakowicz et al.*,*2017* [40]	Case series	23	13 (56.5%)	40.9		22.4	28.4	15: 8	12 (52.17%)	3.020
*Kang et al.*,*2016* [50]	Prospective cohort	20	8 (40%)	38.85		16.63	23.4	17: 3	13 (65%)	4.3
*Kanner et al.*,*2022* [51]	Retrospective cohort	48	24 (50%)	43		50	25.7	34: 14	25 (52.1%)	4.58
*Kim et al.*,*2022* [52]	Retrospective cohort	30	17 (56.7%)	36.6		23.04	N/A	24: 6	15 (50%)	N/A
*landazuri et al.*,*2020* [43]	Prospective cohort	34	16 (47.1%)	35.1		12	N/A	34:60	N/A	1.5
*Le et al.*,*2018* [53]	Prospective cohort	30	14 (47%)	43.3		21.5	26	22: 7	17 (57%)	N/A
*Mo et al.*,*2023* [54]	Retrospective cohort	33	15 (45.4%)	N/A		N/A	10.0	N/A	17 (51.5%)	N/A
*Niu et al.*,*2023* [55]	Retrospective cohort	19	13 (68.4%)	18.1		12	N/A	5: 14	N/A	2.916
*Petito et al.*,*2018* [56]	Retrospective cohort	32	17 (53.13%)	46.97		21.7	N/A	25: 7	22 (68.75%)	N/A
*Sun et al.*,*2024* [57]	Retrospective cohort	27	8 (29.6%)	38.8		12 and 36	N/A	15: 12	8 (29.63%)	N/A
*Tao et al.*,*2017* [58]	Prospective cohort	21	9 (42.86%)	40		12	22	11: 10	10 (47.62%)	2.9
*Vakharia et al.*,*2018* [59]	Retrospective cohort	25	12 (48%)	41.4		29.8	24.4	25: 0	24 (96%)	3.259
*Willie et al.*,*2014* [60]	Clinical trial	13	6 (46.15%)	32.6		14	N/A	9: 4	6 (53.85%)	5.3
*Wu et al.*,*2019* [61	Retrospective cohort	234	110 (47%)	42		30	N/A	170: 64	136 (58.1%)	N/A
*Yossofzai et al.*,*2022* [62]	Retrospective cohort	185	95 (51.35%)	11.3		12	N/A	32: 153	N/A	N/A
*Youngerman et al.*,*2018* [63]	Retrospective cohort	30	12 (40%)	43.5		20.5	19.5	18: 12	11 (36.66%)	7.11
*Youngerman et al.*,*2023* [42]	Retrospective cohort	268	124 (46.3%)	42.33		46.33	N/A	195: 73	150 (56.0%)	N/A

n: number; HS: hippocampal sclerosis

**Table 2:** Baseline characteristics of included studies (RFA, SRS)

Study ID	Study design	Sample size	Male (*n* %)	Mean age (years)	Mean follow up (months)	Mean epilepsy duration (years)	HS: non-HS	Left side involved (*n* %)	Mean ablation volume (cm^3^)
***Radiofrequency thermocoagulation (RFA)***									
*Fan al.*,*2019* [35]	Case series	21	12 (57.14%)	26.1	12	13.9	N/A	11 (52.4%)	N/A
*Fu al.*,*2023* [64]	Retrospective cohort	31	14 (45.16%)	41.29	34.7	22.3	N/A	20 (64.5%)	N/A
*Lee al., 2018* [9]	Clinical trial	7	3 (33.3%)	42.7	12.6	24.49	N/A	5 (71.43%)	0.348
*Li al.*, *2023* [65]	Retrospective cohort	28	16 (57.14%)	25.7	60	11.6	28: 0	16 (57.1%)	N/A
*Moles al.*,*2018* [44]	Retrospective cohort	21	9 (42.86%)	31.3	12	17.8	11: 10	N/A	N/A
*Vojtěch al.*,*2014* [30]	Retrospective cohort	61	31 (50.82%)	41.3	63.6	24.5	N/A	41 (67.2%)	N/A
*Wu al.*,*2014* [66]	Clinical trial	7	3 (42.86%)	29.6	44.6	14	7: 0	4 (57.14%)	N/A
*Zhao al.*,*2017* [67]	Retrospective cohort	12	11 (91.67%)	29.17	34.08	16.67	N/A	N/A	0.4757
***Stereotactic radiosurgery (SRS)***									
*Barbaro al.*,*2009* [32]	RCT	30	12 (40%)	34.1	30	N/A	30: 0	15 (50%)	N/A
*Barbaro al.*,*2018* [34]	RCT	31	14 (45.16%)	16.31	24–36	22.85	31: 0	N/A	6.5
*Hoggard al.*,*2008* [41]	Case series	8	5 (62.5%)	38	24	25	8: 0	N/A	6.2
*Kawamura al.*,*2012* [31]	Retrospective cohort	11	1 (9.09%)	51.5	48	N/A	11: 0	8 (72.7%)	10.6
*Quigg al.*,*2018* [33]	RCT	31	N/A	13	24	26	N/A	N/A	N/A
*Rheims al.*,*2008* [68]	Retrospective cohort	15	6 (40%)	38.5	60	24.9	14: 15	8 (53.3%)	6.96
*Usami al.*,*2012* [36]	Case series	7	2 (28.57%)	33.8	N/A	15.7	6: 7	1 (14.3%)	9.86
*Vojtěch al.*,*2009* [70]	Retrospective cohort	14	6 (42.86%)	33.4	43.5	23.2	14: 0	9 (64.28%)	6.764
*Wang al.*,*2017* [71]	Retrospective cohort	37	21 (56.76%)	29.4	N/A	9.2	37: 0	15 (40.5%)	5.31

n: number; HS: hippocampal sclerosis

### Risk-of-bias assessment and publication bias

Overall, 32 studies were non-randomized, 3 were RCTs and 7 were case series. Using MINORS tool for non-randomized studies, the mean (range) score for non-comparative studies was 11.6 [6–16], and 17.7 [13–21] for comparative studies. All non-randomized studies were considered of moderate methodological quality except 2 were considered poor [30, 31]. Using ROB-2 tool for RCTs, two studies were judged as low risk of bias [32, 33], and one study had some concerns [34]. Using JBI tool for case series, 3 studies were of moderate quality [35–37] and the other 4 were of high quality [38–41]. Tables and figures for the three quality assessment tools are included in ***Online Resource 4.*** Using funnel plots, no significant publication bias was observed for the efficacy and safety outcomes.

### Seizure freedom

**Fig. 2:**
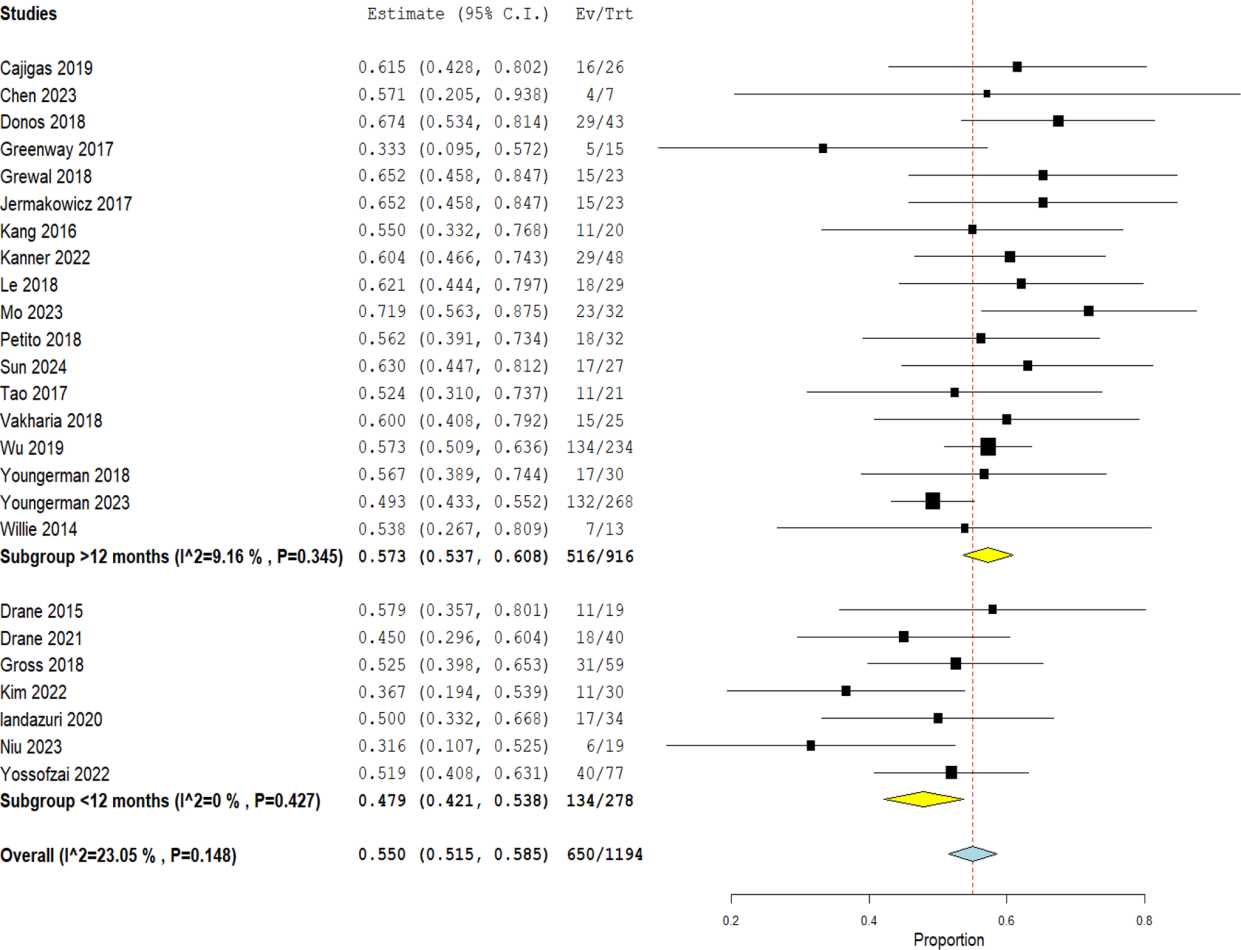
MRgLITT overall rate of Engel I outcome with a subgroup based on follow up period

The rate of overall seizure freedom (Engel I) for MRgLITT was 55.0% (CI 51.5 − 58.5%; I^2 = 23.05%, *P* = 0.148). This was further sub-grouped according to the follow up period to less than or equal 12 months and more than 12 months. The subgroup with follow up period of less than or equal 12 months included 7 studies and had Engel I of 47.9% (CI 42.1 − 53.8%; I^2 = 0%, *P* = 0.427) while the other subgroup with a follow up period of more than 12 months included 18 studies ranging from 13.4 to 50 months had an Engel I of 57.3% (CI 53.7 − 60.8%; I^2 = 9.16%, *P* = 0.345) Fig. 2.

**Fig. 3:**
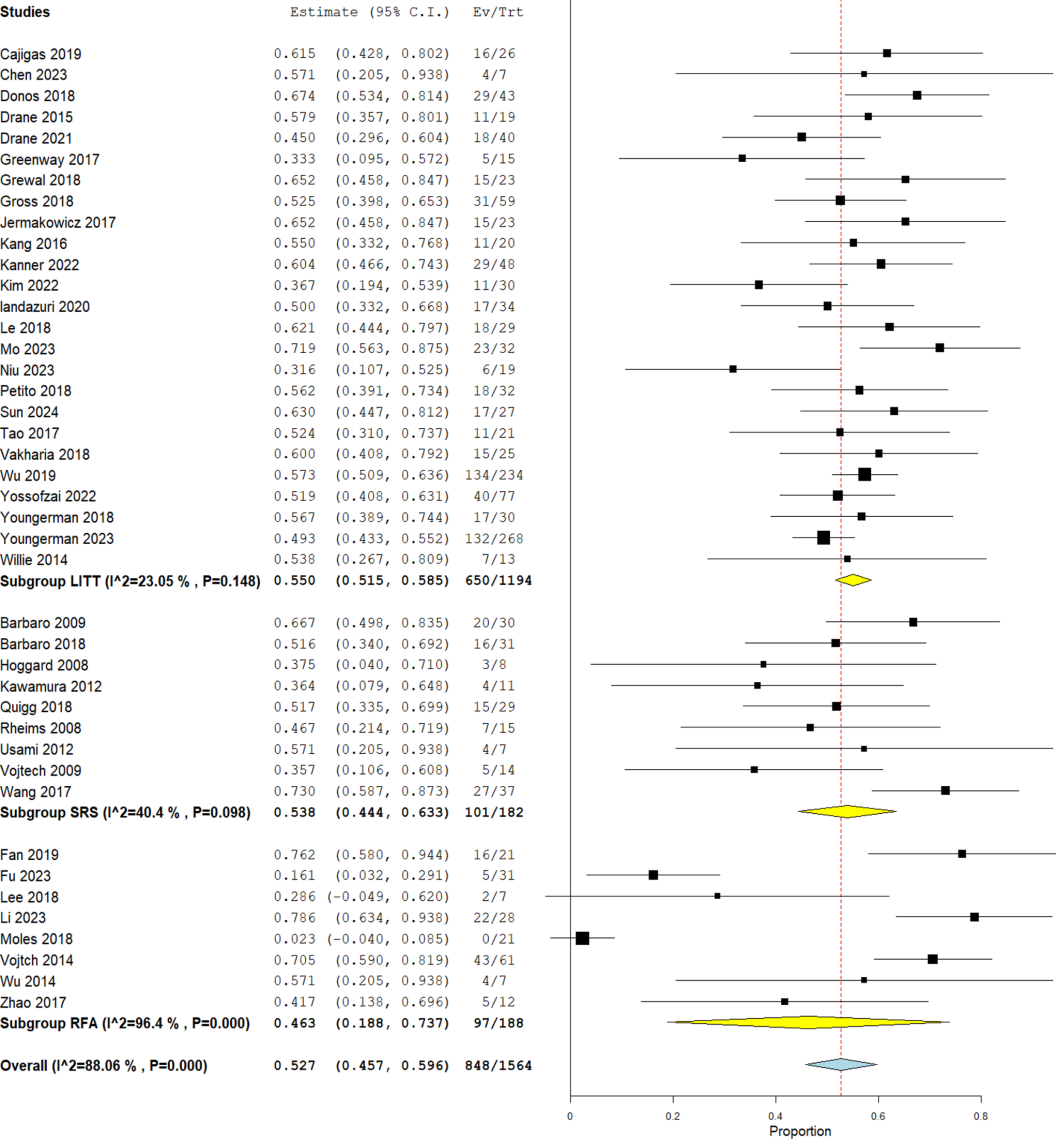
Overall rate of Engel I outcome for the three techniques

MTgLITT yielded the best outcome when compared to SRS which had a rate of Engle I of 53.8% (CI 44.4 − 63.3%; I^2 = 40.4%, *P* = 0.098) and RFA which had 46.3% (CI 18.8 − 73.7%) with considerable heterogeneity (I^2 = 96.4%, *P* = 0.000) Fig. 3.

**Fig. 4:**
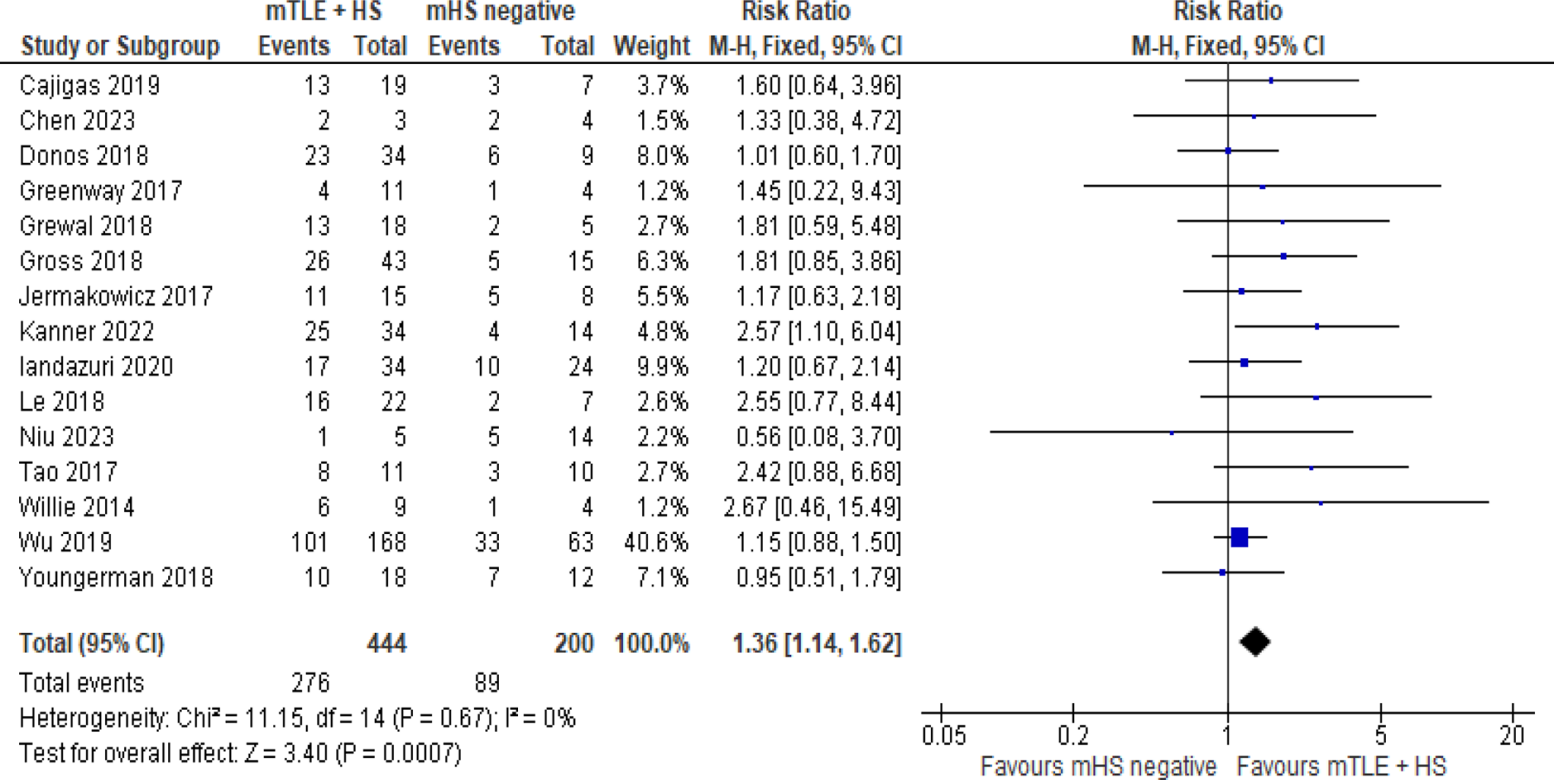
Comparison between Engel I outcome for mTLE patients with or without HS

Higher rates of seizure freedom were found in patients with mTLE associated with HS compared to patients without HS when using MRgLITT; RR 1.36 (CI 1.14–1.62; *P* = 0.0007) Fig. 4.

### Major complications rate

**Fig. 5:**
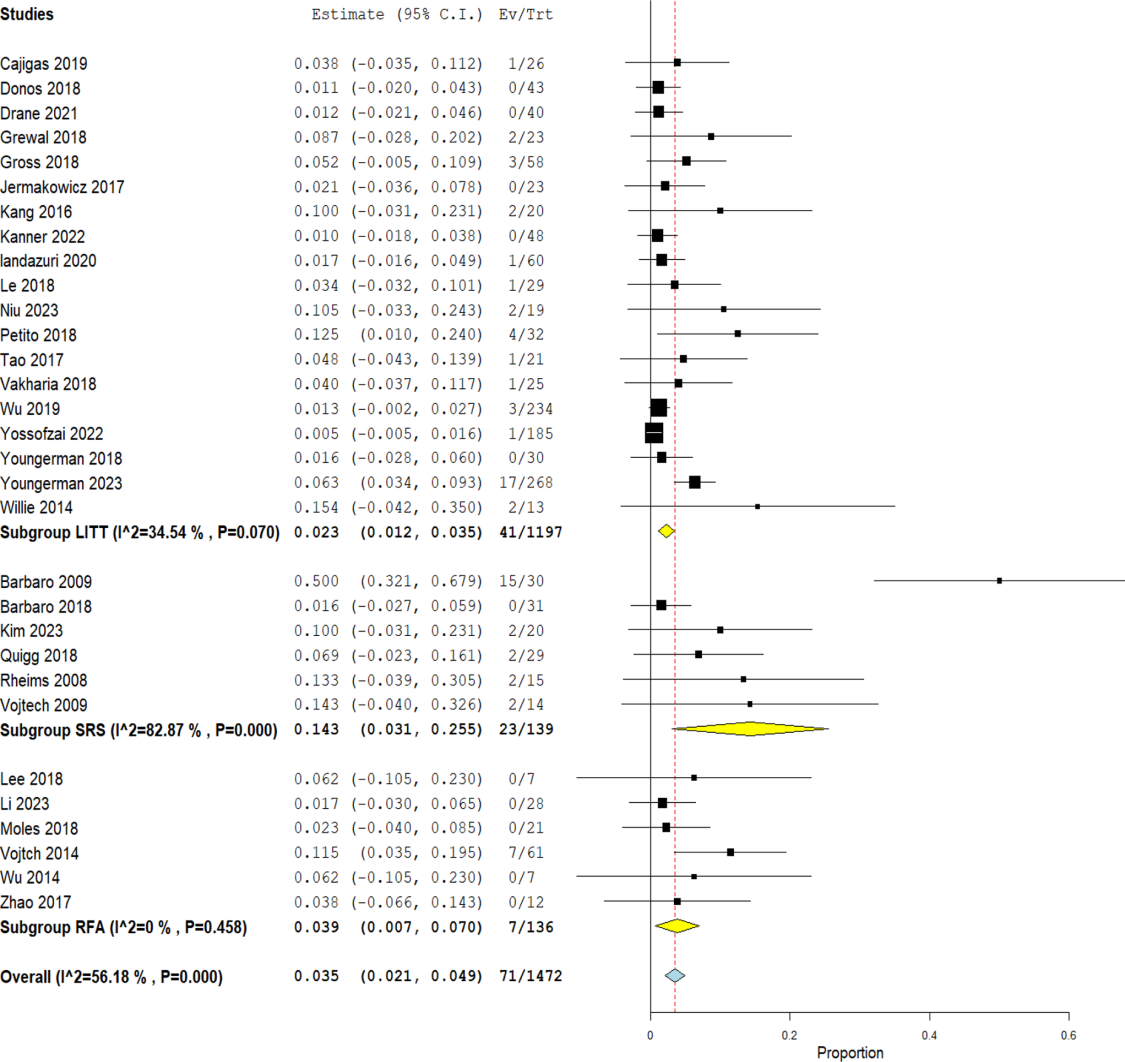
Rate of major complications among the three techniques

The overall rate of major complications for the three techniques was 3.5% (CI 2.1 − 4.9%; I^2 = 56.18%, *P* = 0.000). When comparing the three techniques, MRgLITT was found to be associated with the least rate of major complications (2.3%, CI 1.2 − 3.5%), compared to RFA (3.9%, CI 0.7 − 7.0%; I^2 = 0%, *P* = 0.458) and SRS (14.3%, CI 3.1 − 25.5%) with considerable heterogeneity (I^2 = 82.87%, *P* = 0.000) Fig. 5. MRgLITT had mild heterogeneity (I^2 = 34.54, *P* = 0.070) that was best resolved, after conducting a sensitivity analysis, by exclusion of Youngerman et al. [42] (I^2 = 0%, *P* = 0.492) with an overall effect of 1.3% (CI 0.5 − 2.0%).

### Reoperation

**Fig. 6:**
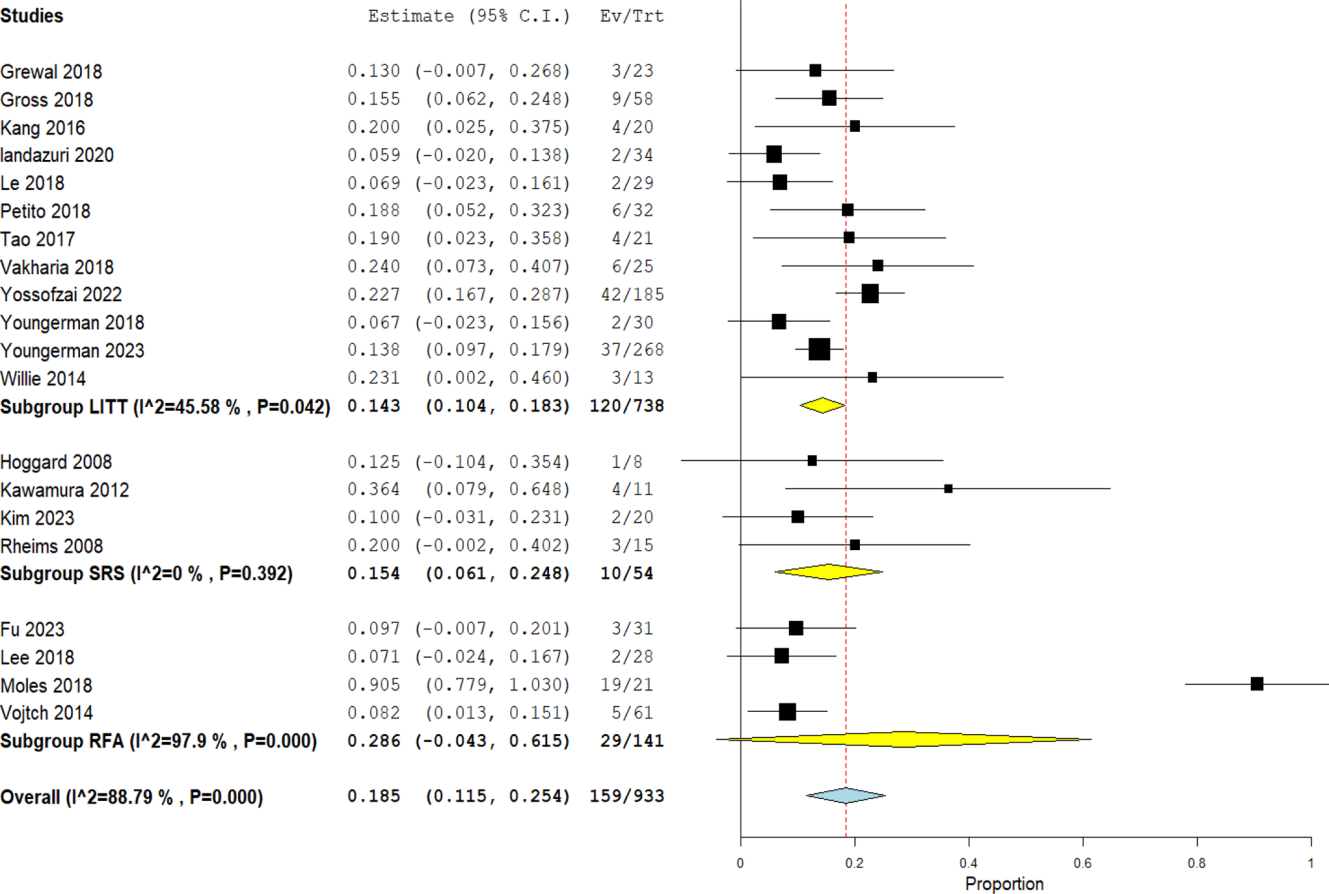
Rate of reoperations among the three techniques

The incidence of reoperation was least with MRgLITT (14.3%, CI 10.4 − 18.3%), higher with SRS (15.4%, CI 6.1 − 24.8%; I^2 = 0%, *P* = 0.392) and highest with RFA (28.6%, CI -4.3 − 61.5%) Fig. 6. MRgLITT had moderate heterogeneity (I^2 = 45.58%, *P* = 0.042) that was best resolved, after conducting a sensitivity analysis, by exclusion of Landazuri et al. [43] (I^2 = 34.91%, *P* = 0.119) with an overall effect of 15.4% (CI 11.6 − 19.2%). RFA had considerable heterogeneity (I^2 = 97.9%, *P* = 0.000) that was best resolved by exclusion of Moles et al. [44] (I^2 = 0%, *P* = 0.94) with an overall effect of 8.2% (CI 3.3 − 13.2%). Figures for sensitivity analyses are included in ***Online Resource 5***.

### Meta-regression analysis

A meta regression analysis was done to compare a number of variables with Engel I outcome to figure if there was a significant correlation; however, all were insignificant. It was done for age (*P* = 0.923), epilepsy duration (*P* = 0.319), ablation volume (*P* = 0.782), left side involvement (*P* = 0.879), and HS association (*P* = 0.575). Graphs of these analysis are provided in ***Online Resource 6***.

## Discussion

Although resective surgeries are the mainstay of treatment of intractable mTLE, there has been a growing interest towards the ablative techniques owing to the fact that they are minimally invasive so are more preferred by patients, having potentially less adverse effects than open procedures and serving patients who are contraindicated for open surgeries [71]. It was of importance to compare the efficacy, risk of major complications and reoperations between the most used ablative techniques to know which one is more established and point out the need of further improvement in the others to reach maximal efficacy possible from all techniques.

### Seizure outcome

When comparing Engel I outcome between the three techniques Fig. 3, it was found that MRgLITT had the highest rates (55.0%) which might be due to the fact that a significantly larger number of studies have been done on that technique leading to faster improvements being made, whether in the methodology and precision or in the more careful selection of patients who will benefit from the technique, compared to the other two. SRS has lower rate of Engle I (53.8%) which could be attributed to the delayed effect of this technique on the seizure outcome which can be from 18 to 24 months according to Hoggard et al. [41] and up to 53 months in Rheims et al. [67] which suggests more patients could have Engel I outcome but not documented by the studies as they occurred after the study period, besides having intolerable patients who are yet to achieve seizure freedom undergo open surgery for seizure control and do not wait for the technique’s result making it difficult to assess effectiveness [31].

RFA represents the lowest outcomes (46.3%) and the highest heterogeneity which could be attributed to two reasons. Firstly, the total number of patients in most studies of LITT or SRS are close to each other; however, it is very variable in the studies of RFA. Secondly and a more important reason is the different protocols for RFA. For instance, in MRgLITT the same way of application is utilized in almost all studies where a visualase fiber probe is stereotactically guided through an occipital trajectory into the area to be ablated [37, 38, 47, 51, 58]. Conversely, for RFA in Lee et al., Vojtech et al., and Fu et al. [9, 30, 64] they used a single radiofrequency lead/probe through a single trajectory like in MRgLITT, while in Moles et al., and Wu et al. [44, 65] the radiofrequency waves were transmitted by a generator system through the electrodes already inserted at different points to initially take an EEG reading, and in Fan et al., and Li et al. [35, 65] they delivered the waves to one, two contiguous (on same plane), or multiple cross-connection electrodes to either ablate an area or cut connections between areas. They also differed in the trajectory they used where Lee et al., Vojtech et al., and Fu et al. [9, 30, 64] used occipital trajectory, Moles et al. [44] used a transtemporal (orthogonal) approach, and Wu et al., and Zhao et al. [66, 67] used a transfrontal approach. Despite the studies using the same technique, the different methodologies will make it harder to use the results for comparison especially with the low number of papers. Moreover, the use of different trajectories rather than the occipital, which is along the hippocampal axis, together with the relative smaller size of electrodes compared to a probe, may limit the amount of tissue ablated, and lead to less effective outcomes [44].

Since MRgLITT had the largest number of studies with abundant data and the best rate of Engel I outcome, two other separate analyses were made on this technique. Analysis of Engel I outcome was made based on a subgroup of mean follow up periods: one being less than or equal 12 months and the other being more than 12 months Fig. 2. A higher rate of Engel I was associated with the subgroup of more than 12 months follow up (57.3% vs. 47.9%) and is contradictory to what is usually described in studies where if there was to be a change along time in the seizure outcome it would be patients dropping to a lower level of Engel not increasing [37, 38]. With HS being the most common cause of mTLE [3], another analysis was made to see if the seizure outcome differs between patients having mTLE with HS compared to HS negative patients Fig. 4. Risk ratio was 1.36 (CI 1.14–1.62, *P* = 0.0007) favoring mTLE with HS which indicates that using this technique in a patient with HS is more effective and will yield a better rate of Engle I outcome than in patients without HS. On the contrary, other studies stated that although patients with Engle I was more in HS group than HS negative, there was no statistically significant difference between the two groups [37, 38].

### Major complications

The rate of major complications was compared among the three techniques Fig. 5. It was highest with SRS (14.3%) and lowest with MRgLITT (2.3%). Event number in each of MRgLITT, RFA and SRS were 41/1197, 7/136, and 23/139, respectively. The most common complication was VFDs mainly in the form of homonymous hemianopias or quadrantanopias. Second most common was ICH in the form of epidural, subdural or intracerebral bleeding and only one case with hemocephalus reported in Vojtech et al. [30]. Other complications were scarce in number and included infection, meningitis, and pulmonary embolism. Rate is close between MRgLITT and RFA; however, it is substantially higher for SRS and this could be explained by multiple reasons. Hoggard et al. [41] explained that vasogenic edema occurs at 6–12 months period following the procedure, which is inside the mean follow up period for complications (mean of 24 months). This edema although subsides with the use of corticosteroids, it was found by postmortem histological examination of one of the patients that it leads to neuronal loss and extensive gliosis which could be a factor for further complication development. A test dose/temperature is first used before delivering the target ablative dose to test proper placement of leads/electrodes for both MRgLITT and RFA techniques [9, 38] which is a step lacking in the protocol of SRS that may lead to more complications. According to the data available, we could calculate the mean ablated volumes for both MRgLITT and SRS yielding 4.28 and 7.46, respectively which is significantly higher in SRS than MRgLITT. In a study, they explain that high dose of radiation or increased target volume not only increases the risk of acute radiation effect but also delayed radiation necrosis that may develop over few months or years [31]. We discovered the high heterogeneity in the results for SRS is related to the high rate of complication in Barbaro et al. study [32]. They mentioned that radiosurgery lesions have a “superselective” nature when they explained why their results with verbal memory were better compared to other studies; this also could possibly explain why they had a high rate VFDs compared to the other studies included in this systematic review.

### Reoperations

The rate of reoperations was also measured for the three techniques Fig. 6. Reoperations were either in the form of repeat ablation with same technique or resective surgery and in some cases, patients underwent vagal nerve stimulation or responsive neurostimulation [42]. Lowest rates were with MRgLITT (14.3%) which is consistent with the results of Engle I outcome. Risk of reoperation did not only depend on the effectiveness of the technique but also on the ablated volume where in Gross et al. [49] they opted for repeat ablations in three patients when they found remnant unablated lesions were responsible for the seizures hence they used a different trajectory to target those remnants and the patients achieved Engle I outcome. They demonstrated in the same study that two other patients had to undergo ATL after repeat ablations as it was ineffective in eliminating seizures which implies that it also depends on patient’s factors. Reoperation rate was higher in SRS (15.4%) which could be explained by the delayed seizure elimination by this technique leading some patients who cannot tolerate seizures to undergo reoperations [31]. Rate was highest for RFA (28.6%) which could be due to the fact that different approaches were used in different studies, which would also limit our ability to generalize this percentage for the technique; for this reason, more RCTs need to be made on the more successful protocols to better understand the effectiveness of the technique. The cause of heterogeneity in this technique’s result is the high rate in Moles et al. [44] which is attributed to the fact that none of the patients achieved seizure freedom so most of the patients needed ATL.

### Limitations

A major limitation in this study is that all the included data for each technique is derived from single arm studies as there are no studies available that compare between those techniques in a double arm fashion and to the best of our knowledge, this is the first meta-analysis to be done to compare between those three techniques. Another issue was that most studies included a small sample size, so large RCTs and prospective cohort studies are required to validate our results. Not only the small sample sizes but also the small number of studies especially in RFA and SRS and the different protocols used have increased the heterogeneity in some of the outcomes. Additionally, there was no standardization among studies neither in the follow up periods for assessing Engel outcome nor during assessment of complication severity hence we attempted to define clearly how we used these variable data to make our analysis more reliable.

## Conclusion

In the light of the data presented in this paper, Engel I results were comparable between the three techniques; however, MRgLITT seems to be a more promising technique owing to it having the highest seizure freedom outcome, and the lowest rates of major complications and reoperations. More RCTs and prospective cohort studies are required on both RFA and SRS to better assess their efficacy and further improve the techniques. Since all of the three techniques are relatively novel procedures, they will continue to benefit from the increasing learning curve of surgeons, adaptation of ablation techniques, and improvement of patient selection. Additionally, clinical trials comparing the techniques together are needed for a more effective comparison. It is important to keep in mind that the choice of one of the techniques should not only depend on its efficacy but also tailored according to the patient’s case, considering clinical and radiological differences, and preferences.

## Electronic supplementary material

Below is the link to the electronic supplementary material.

Supplementary Material 1

Supplementary Material 2

Supplementary Material 3

Supplementary Material 4

Supplementary Material 5

Supplementary Material 6

## Data Availability

No datasets were generated or analysed during the current study.

## Declarations

### Prospero registery number

CRD42024594624.
