# Supplementary material for: Efficacy and safety of laser interstitial thermal therapy versus radiofrequency ablation and stereotactic radiosurgery in the treatment of intractable mesial temporal lobe epilepsy: a systematic review and meta-analysis
Source: Neurosurg Rev. 2025 Jan 21;48(1):71. doi: 10.1007/s10143-025-03215-8 (PMC11750889; doi:10.1007/s10143-025-03215-8)
Supplement: Supplementary file 1 — Supplementary Material 1 [file 10143_2025_3215_MOESM1_ESM.docx]

**Laser interstitial thermal therapy versus radiofrequency ablation and stereotactic radiosurgery in the treatment of intractable mesial temporal lobe epilepsy: A systematic review and meta-analysis**

Youstina Mohsen^1^, Khalid Sarhan^2^, Ibrahim Saleh Alawadi^1^, Reem Reda Elmahdi^1^, Yasmeena Abdelall Kozaa^1^, Menna A. Gomaa^1^, Ibrahim Serag^2^, Mostafa Shahein^3^

^1^Mansoura Manchester Program for Medical Education (MMPME), Faculty of Medicine, Mansoura University, Mansoura, Egypt

^2^Faculty of Medicine, Mansoura University, Mansoura, Egypt

^3^Department of neurosurgery, Faculty of Medicine, Mansoura University, Egypt

CORRESPONDING AUTHOR:

Youstina Mohsen

Email: [youstinamohsen1@std.mans.edu.eg](mailto:youstinamohsen1@std.mans.edu.eg), [youstinamosensamir@gmail.com](mailto:youstinamosensamir@gmail.com)

ORCID: 0000-0002-5949-1794

Submitted to Neurosurgical Review journal

***Search Strategy***

("epilepsy" or "epileptic" or "temporal epilep*" or "TLE" or "mesial temp* epilep*" or "mesial temporal lobe epilepsy" or "mTLE" or "epileptic") AND ("Stereotactic radiosurgery" or "radiosurgery" or "Gamma Knife" or "Gamma Knives" or GammaKnife or "GK RS" or GKRS or "GK-RS" or "laser abla*" or "laser ablation" or "thermal ablati*" or "MR guided laser" or "MRgLITT" or "LITT" or "laser induced therm*" or "laser" or "laser interstitial" or "laser interstiti* ther*" or "laser interstitial thermal therapy" or "thermal therap*" or "RFA" or "radiofrequency ablation" or "radiofrequency therm* ablat*" or "radio frequency ablation" or "stereotactic ablation" or "stereotac* coagulation" or "SEEG radiofre*" or "stereotactic radiofrequen*" or "SEEG guided radiofrequency ablation" or "radiofrequency coagulation" or "radiofrequency thermocoagulation" or "radiofrequency thermo*")

1. ("epilepsy" or "epileptic" or "temporal epilep*" or "TLE" or "mesial temp* epilep*" or "mesial temporal lobe epilepsy" or "mTLE" or "epileptic")

2. ("Stereotactic radiosurgery" or "radiosurgery" or "Gamma Knife" or "Gamma Knives" or GammaKnife or "GK RS" or GKRS or "GK-RS")

3. ("laser abla*" or "laser ablation" or "thermal ablati*" or "MR guided laser" or "MRgLITT" or "LITT" or "laser induced therm*" or "laser" or "laser interstitial" or "laser interstiti* ther*" or "laser interstitial thermal therapy" or "thermal therap*")

4. ("RFA" or "radiofrequency ablation" or "radiofrequency therm* ablat*" or "radio frequency ablation" or "stereotactic ablation" or "stereotac* coagulation" or "SEEG radiofre*" or "stereotactic radiofrequen*" or "SEEG guided radiofrequency ablation" or "radiofrequency coagulation" or "radiofrequency thermocoagulation" or "radiofrequency thermo*")
