# Supplementary material for: Efficacy and safety of laser interstitial thermal therapy versus radiofrequency ablation and stereotactic radiosurgery in the treatment of intractable mesial temporal lobe epilepsy: a systematic review and meta-analysis
Source: Neurosurg Rev. 2025 Jan 21;48(1):71. doi: 10.1007/s10143-025-03215-8 (PMC11750889; doi:10.1007/s10143-025-03215-8)
Supplement: Supplementary file 2 — Supplementary Material 2 [file 10143_2025_3215_MOESM2_ESM.docx]

**Laser interstitial thermal therapy versus radiofrequency ablation and stereotactic radiosurgery in the treatment of intractable mesial temporal lobe epilepsy: A systematic review and meta-analysis**

Youstina Mohsen^1^, Khalid Sarhan^2^, Ibrahim Saleh Alawadi^1^, Reem Reda Elmahdi^1^, Yasmeena Abdelall Kozaa^1^, Menna A. Gomaa^1^, Ibrahim Serag^2^, Mostafa Shahein^3^

^1^Mansoura Manchester Program for Medical Education (MMPME), Faculty of Medicine, Mansoura University, Mansoura, Egypt

^2^Faculty of Medicine, Mansoura University, Mansoura, Egypt

^3^Department of neurosurgery, Faculty of Medicine, Mansoura University, Egypt

CORRESPONDING AUTHOR:

Youstina Mohsen

Email: [youstinamohsen1@std.mans.edu.eg](mailto:youstinamohsen1@std.mans.edu.eg), [youstinamosensamir@gmail.com](mailto:youstinamosensamir@gmail.com)

ORCID: 0000-0002-5949-1794

Submitted to Neurosurgical Review journal

| ***Supplementary 3: Summary of included studies for RFA and SRS*** | | | | | | |
| --- | --- | --- | --- | --- | --- | --- |
| *Study ID* | *Country* | *Duration* | *Inclusion criteria* | *Exclusion criteria* | *Outcomes* | *Main findings* |
| \| *Radiofrequency ablation (RFA)* \| \| --- \| | | | | | | |
| *Fan al., 2019* | China | 2016 - 2017 | - DR mTLE, HS patients | - Patients with distinct lesions such as tumor, cavernous malformation, arteriovenous malformation, infarction, and normal hippocampi, suspected extrahippocampal or bilateral origination of seizures, dual pathology. | **1ry:** evaluate the postsurgical seizure outcome using Eagle classification following SEEG guided RF-TC for mesial temporal lobe epilepsy with HS.  **2ry:** postablation complications. | Optimized SEEG-guided RF-TC is a promising complementary option for the treatment of MTLE-HS |
| *Fu al., 2023* | Taiwan | 2016 - 2020 | - mTLE patients - underwent limited hippocampal RFTC | - N/A | **1ry:** long-term seizure control and the neuropsychologic outcomes of limited hippocampal RFTC in patients with DRE due to mTLE and assess the prognostic factors of long term seizure control in these patients.  **2ry:** Neuropsychology performance. | Limited hippocampal RFTC is a safe stereotactic minimal invasive procedure with good neuropsychologic outcome and acceptable efficacy of seizure reduction in appropriately selected patients. |
| *Lee al., 2018* | Taiwan | N/A | - Unilateral DR-MTLE patients - able to keep a reliable seizure diary - a prospective seizure frequency of at least two complex partial seizures per month or secondary generalized tonic-clonic seizures during a baseline of 3 months | - Underwent multiple seizure surgeries - Have poor recording of seizure frequency before the RFTC surgery | **1ry:** Seizure outcome by using the Engel classification. **2ry:** Memory function and IQ by using Wechsler Adult Intelligence Scale. Changes in the imaging studies and EEG pat- terns postoperatively. | Two patients achived Engle I, another two patients achived Engle II, and three patients achived Engle III. No significant change in memory function and IQ after the procedure. |
| *Li al., 2023* | China | 2016 - 2018 | - Confirmed diagnosis of HS - Clinically consistent EEG manifestations originating from the hippocampus - A minimum post-operative follow-up of 60 months | - Incomplete follow-up data - Carrying out other brain procedures within the follow-up period that might affect the results | Seizure outcomes using Engel classification and complications. | SEEG-3D RFTC is a promising alternative for patients with MTLE-HS due to its long-term efficacy. |
| *Moles al., 2018* | France | 2001-2016 | - confirmed TLE with SEEG - Patients for whom the data from non-invasive investigations were not sufficiently congruent to allow direct resective surgery without performing invasive recordings with SEEG | - N/A | **1ry:** seizure freedom at 1 year.  **2ry:** response (at least 50% reduction of seizure frequency) at 1 year. | SEEG-guided RF-TC is not as effective as ATL in TLE. only patients with dominant mesial involvement for whom hippocampectomy is not an option. SEEG guided RF-TC sould be of benefit for them as no memory impairment results from it. |
| *Vojtěch al., 2014* | Czech Republic | 2004 - 2010 | - Patients underwent SAHE | - N/A | **1ry:** to assess the long-term seizure outcome after SAHE in a larger group of patients with MTLE.  **2ry:** postablation early and late complications. | Evidence for good long-term seizure outcomes after SAHE. SAHE could be an alternative therapy for mTLE. |
| *Wu al., 2014* | China | N/A | - Aged ≥15 years‐old - Experienced simple and/or complex partial seizures with or without secondary generalization - Experienced ≥3 complex partial seizures during the 3 months (12‐week) baseline seizure diary period, with ≥1 seizure occurring within the last 2 months - Electrographic evidence of seizures arising from one temporal lobe, with radiographic evidence of mesial temporal sclerosis in the same temporal lobe. | - Patients with normal magnetic resonance imaging scans, bilateral hippocampal damage or cortical lesions | Treatment outcomes were evaluated by computed tomography scans and Engel classification criteria. | Robot‐assisted frameless stereotaxy for deep electrode implantation and RFTC is indicated to be a safe and effective method that may be used effectively in clinical practice. |
| *Zhao al., 2017* | China | 2006 - 2011 | - Took more than two kinds of antiepileptic drugs for at least two years - Undergone clinical follow-up for at least one year - Completed pre- and postoperative evaluation for seizure outcome, including Engel’s classification and the Liverpool Seizure Severity Scale (LSSS) - Undergone preoperative (3-7 days) and postoperative (six months) neuropsychological examinations. | - N/A | To explore the safety and efficacy of bilateral trans frontal minimal  RFTC of the amygdalohippocampal complex for the treatment of bilateral mTLE. | Bilateral transfrontal minimal RFTC of the amygdalohippocampal complex may terminate seizures or reduce seizure severity in patients with bilateral mTLE. |
| \| *Stereotactic radiosurgery (SRS)* \|  \|  \| \| --- \| --- \| --- \| | | | | | | |
| *Barbaro al., 2009* | USA | N/A | - Unilateral mTLE as defined by presurgical evaluation including neurological history and ex- amination, routine interictal electroencephalogram (EEG), ictal video-EEG, magnetic resonance images (MRIs), neuro- psychological evaluations, and intracarotid sodium amytal (Wada) tests. | - Patients had medically intractable partial seizures with an average of at least three complex partial seizures per month over a 3-month pretreatment observation phase - Any other radiological abnormalities, diabetes, hypertension, or pregnancy (as radi-ation safety precautions). - Psychological diagnoses that would make it difficult to accurately assess seizures, significant medical (including neurological) comorbidities, and poorly compliant or drug-abusing patients. | Seizure diaries evaluated the final seizure remission between months 24 and 36. Verbal memory was evaluated at baseline and 24m with the Wechsler Memory Scale–Revised (WMS-R) and California Verbal Learning Test (CVLT). Patients were classified as having “significant improvement,” “no change,” and “significant impairment” based on relative change indices. postoperative complications. | RS for unilateral mesial temporal lobe epilepsy offers seizure remission rates comparable with those reported previously for open surgery. There were no major safety concerns with high-dose RS compared with low-dose RS. |
| *Barbaro al., 2018* | USA, UK, India | N/A | - ≥18 years old - Documented 3 months during which at least 3 focal-onset seizures with impairment of consciousness occurred during stable anticonvulsant administration - Lacked neurological or visual deficits that would confound follow-up - Eligible for ATL to treat pharmaco- resistant unilateral mTLE. | - Pregnancy - Supratentorial MRI abnormalities - Diabetes mellitus - Use of vigabatrin - Psychiatric diagnoses that would make it difficult to accurately assess seizures - Significant comorbidities - Poor compliance - Current drug abuse | **1ry:** compare SRS versus ATL for patients with pharmacoresistant unilateral mTLE.  **2ry:** seizure remission, verbal memory (VM), and quality of life (QOL). | The data suggest that ATL has an advantage over SRS in terms of proportion of seizure remission, and both SRS and ATL appear to have effectiveness and reasonable safety as treatments for mTLE. SRS is an alternative to ATL for patients with contraindications for or with reluctance to undergo open surgery. |
| *Hoggard al., 2008* | England | 1998 - 2000 | - >18 years of age - Offered epilepsy surgery for complex partial seizures that were resistant to medication. | - Patients with other or additional abnormalities evident on the MRI scans, such as a mass lesion or cortical dysplasia | Analyzing the MRI and spectroscopic changes that follow the treatment and report the clinical sequelae of the procedure. | There are marked changes in MRI scans and magnetic resonance spec troscopic findings after patients undergo radiosurgery for temporal lobe epilepsy. initial findings suggest that some patients may have a period of distressing symptoms that accompany changes that are visualized on the MRI scans. |
| *Kawamura al., 2012* | Japan | 1997 - 2000 | - Medically intractable complex partial seizures with or without secondary generalization - Gave written informed consent after receiving explanations of the diagnosis of intractable mTLE, and the rationales and the usual procedures for determining suitability for GKRS. | - N/A | The long-term seizure outcome and adverse effects of GKRS for mTLE in 11 patients after follow-up periods of over 9 years. | GKRS for mTLE causes adverse effects of delayed seizure remission and symptomatic radiation- induced complications. Therefore, GKRS cannot be considered as an ideal alternative to surgery for mTLE. Long-term follow-up studies including MR imaging with contrast medium are required for the patients even after successful control of seizures. |
| *Quigg al., 2018* | US, UK, and India | NR | - ≥ 18 years old - had pharmaco-resistant unilateral mTLE - Eligible for resective epilepsy surgery | - Patients with abnormalities in the pre-treatment visual field assessment | Engel Class and Visual field. | Effective surgery (defined by seizure remission) of the mesial temporal lobe results in about a 90% incidence of typical visual field defects regardless of method. |
| *Rheims al., 2008* | France | 1994 - 2004 | - Presenting with TLE - Localisation of the epileptogenic zone was based on electro-clinical data, including or not intracranial monitoring, and could either be restricted to the mesial temporal structures or involve the temporal neocortex. | - N/A | Seizure outcome was evaluated by ILAE post-surgical outcome scale. Prognostic predictors of seizure outcome and GKS-related MRI changes were assessed. | When a positive outcome is achieved, the risk of seizure recurrence remains low at long term. In patients with typical mTLE, long-term GKS results may be closed to those observed after conventional surgery. Conversely, this procedure should not be proposed to patients in whom SEEG results suggest that the epileptogenic zone is not restricted to mesial temporal structures. |
| *Usami al., 2012* | Japan | 1996 - 1999 | - Diagnosed with TLE who underwent GKS | - N/A | **1ry:** seizure frequency and complications  **2ry:** postoperative changes were evaluated by periodic MRI examinations. | High-dose treatment resulted in sufficient seizure control but carried a significant risk of SRN after several years. Excessive target volume was considered as a reason for delayed necrosis. Drawbacks such as a delay in seizure control and the risk of SRN should be considered when the clinical significance of this treatment is evaluated. |
| *Vojtěch al., 2009* | Czech Republic | 1995 - 1999 | - Patients with LGK SR for intractable mTLE | - N/A | **1ry:** determine the efficacy of gamma knife radiosurgery in the treatment of mesial temporal lobe epilepsy due to mesial temporal sclerosis.  **2ry:** postablation complications and Cognitive outcomes. | Radiosurgery with 25, 20, or 18-Gy marginal dose levels did not lead to seizure control in our patient series, although subsequent epilepsy surgery could stop seizures. Higher doses were associated with the risk of brain edema, intracranial hypertension, and a temporary increase in seizure frequency. |
| *Wang al., 2017* | China | 2011 - 2013 | - Diagnosed as mTLE based on the Brain MRI Check, Scalp EEG and Long-term EEG before surgery - No cerebral trauma within three years after treatment - Informed and agreed to this study and signed informed consent forms | - Patients who suffered from serious substantive organ diseases, such as heart, liver, spleen diseases - Patients accompanied with other neurologic diseases | **1ry:** evaluate the postsurgical seizure outcome using Engle classification, the clinical efficacy of gamma knife and surgery treatment of mTLE and their effects on EF-Tumt and EF-Tsmt expression to provide clinically scientific basis for further improvement of the therapeutic effect of this disease.  **2ry:** postoperative complications. | Both surgery and gamma knife could treat mTLE effectively, and its effect may play a role in reducing the expression of EF-Tsmt and EF-Tumt protein in brain tissue. |

DR: drug resistant; mTLE: mesial temporal lobe epilepsy; HS: hippocampal sclerosis; SEEG: stereoelectroencephalography; RF-TC: radiofrequency thermocoagulation; SAHE: stereotactic radiofrequency amygdalohippocampectomy; RS: radiosurgery; ATL: anterior temporal lobectomy; MRI: magnetic resonance imaging; GKRS: gamma knife radiosurgery; SRN: symptomatic radiation necrosis; LGK SR: Leksell Gamma Knife stereotactic radiosurgery.
