# Supplementary material for: Efficacy and safety of laser interstitial thermal therapy versus radiofrequency ablation and stereotactic radiosurgery in the treatment of intractable mesial temporal lobe epilepsy: a systematic review and meta-analysis
Source: Neurosurg Rev. 2025 Jan 21;48(1):71. doi: 10.1007/s10143-025-03215-8 (PMC11750889; doi:10.1007/s10143-025-03215-8)
Supplement: Supplementary file 3 — Supplementary Material 3 [file 10143_2025_3215_MOESM3_ESM.docx]

**Laser interstitial thermal therapy versus radiofrequency ablation and stereotactic radiosurgery in the treatment of intractable mesial temporal lobe epilepsy: A systematic review and meta-analysis**

Youstina Mohsen^1^, Khalid Sarhan^2^, Ibrahim Saleh Alawadi^1^, Reem Reda Elmahdi^1^, Yasmeena Abdelall Kozaa^1^, Menna A. Gomaa^1^, Ibrahim Serag^2^, Mostafa Shahein^3^

^1^Mansoura Manchester Program for Medical Education (MMPME), Faculty of Medicine, Mansoura University, Mansoura, Egypt

^2^Faculty of Medicine, Mansoura University, Mansoura, Egypt

^3^Department of neurosurgery, Faculty of Medicine, Mansoura University, Egypt

CORRESPONDING AUTHOR:

Youstina Mohsen

Email: [youstinamohsen1@std.mans.edu.eg](mailto:youstinamohsen1@std.mans.edu.eg), [youstinamosensamir@gmail.com](mailto:youstinamosensamir@gmail.com)

ORCID: 0000-0002-5949-1794

Submitted to Neurosurgical Review journal

| ***Supplementary 2:*** ***Summary of included studies for LITT*** | | | | | | |
| --- | --- | --- | --- | --- | --- | --- |
| *Study ID* | *Country* | *Duration* | *Inclusion criteria* | *Exclusion criteria* | *Outcomes* | *Main findings* |
| *Cajigas et al., 2019* | USA | 2013 - 2018 | - mTLE patients - Underwent LITT | - N/A | **1ry:** Postsurgical seizure outcome using Engle classification after a minimum follow-up period of 2 years.  **2ry:** Postablation complications. | LITT appears to be a safe and effective initial surgical option for treatment-resistant mTE. Among patients who have seizures after treatment, those without MTS appear to have seizures earlier than those with MTS. |
| *Chen et al., 2023* | USA | N/A | - Adult mTLE patients - Under-went MRgLITT - With at least - Follow-up > 6 months | - N/A | Investigate the predictive  value of pre-MRgLITT and post-MRgLITT iECoG on seizure outcome  in patients with mTLE. | Intraoperative reduction in epileptiform discharges during mesial temporal lobe MRgLITT may potentially predict seizure outcomes and may serve as an intraoperative biomarker for satisfactory ablation. |
| *Donos et al., 2018* | USA | 2012 - 2017 | - patients diagnosed with either left or right mTLE - Underwent LITT | - N/A | **1ry:** Postsurgical seizure outcome using Engle classification.  **2ry:** Postablation complications and Cognitive outcomes. | Seizure-free outcomes following LITT may be comparable in carefully selected patients with and without MTS, and these outcomes are comparable with outcomes following microsurgical resection. Failures may result from non-mesial components of the epileptogenic network that are not affected by LITT. Cognitive declines following mTL-LITT are modest, and principally affect memory processes. |
| *Drane et al., 2015* | USA | N/A | - ≥ 18 years old - Native English speakers - Left-hemisphere dominant for language (except two) | - <18 years old - not assessed cognitively before undergoing a subsequent open resection | Object recognition and naming outcome with MRI-guided SLAH for temporal lobe epilepsy. | The hippocampus does not appear to be an essential component of neural networks underlying name retrieval or recognition of common objects or famous faces. |
| *Drane et al., 2021* | USA | N/A | - First 40 patients undergoing SLAH and 40 patients undergoing traditional ATL - ≥16 years old - Native English speakers | - Underwent a prior open resection surgery - Refuse to participate in the study - Invalid post-surgical data | To evaluate declarative memory outcomes. | While verbal memory function can decline after laser ablation of the amygdalohippocampal complex, it is better preserved when compared to open temporal lobe resection. Dominant hippocampus is not uniquely responsible for verbal memory. |
| *Greenway et al., 2017* | USA | 2013 - 2016 | - ≥18 years old - Both male and female - DR-mTLE patients - Follow-up > 6 months | - Less than 18 years old - Pregnant - Follow-up < 6 months | **1ry:** Seizure outcomes using Engel classification.  **2ry:** Evaluate the postsurgical Neuropsychological (memory and language) outcomes. | Stereotactic laser ablation can result in clinically significant and meaningful decline in verbal and visual memory when comparing patients to their own presurgical baseline. Naming ability, conversely, is much less likely to be impacted by stereotactic laser ablation and may improve after the procedure. |
| *Grewal et al., 2018* | USA | 2011 - 2015 | - Follow-up of 60 months | - N/A | Engel classification, visual field, neurophysicological test was specific for verbal memory and included Boston Naming Test, Auditory Verbal Learning Test, and the California Verbal Learning Test. | There was no significant correlation between the ablation volume after LITT and seizure outcomes. |
| *Gross et al., 2018* | USA | 2011-2016 | - mTLE patients | - N/A | **1ry:** Surgical outcomes using Engel’s classification.  **2ry:** Postoperative complications, seizure frequency, antiepileptic medication doses, and quality of life. | This novel procedure is an effective minimally invasive alternative to resective surgery. In the minority of patients not free of disabling seizures, laser ablation presents no barrier to additional open. |
| [*Jermakowicz et al., 2017*](https://drive.google.com/open?id=1K7FCPBKD84ub-Os236MIWGf3_o8v-u_D&usp=drive_copy) | USA | N/A | - DR-mTLE patients underwent LITT - Follow-up ≥ 12 months | - N/A | **1ry:** Seizure outcome data (Engel classifications).  **2ry:** WMS-IV Logical Memory I, WMS-IV Logical Memory II, BMVT-R Total Learning, BMVT-R Delayed Recall. | At 1-year follow-up, LITT appears to be a safe and effective tool for the treatment of mTLE, although a longer follow-up period is necessary to confirm these observations. |
| *Kang et al., 2016* | USA | 2011-2014 | - mTLE patients | - N/A | **1ry:** Postsurgical seizure outcome using Engle classification.  **2ry:** Verbal memory outcome. | MRI-guided stereotactic LITT is a safe alternative to ATL in patients with medically intractable mTLE. |
| *Kanner et al., 2022* | USA | 2013 - 2019 | - mTLE patients | - N/A | **1ry:** Postsurgical seizure outcome using Engle classification.  **2ry:** The neuropsychological evaluation included the Mini-Neuropsychiatric Interview and the Beck Depression Inventory-II25 and the Beck Anxiety Inventory. | LITT appears to be a safe and effective surgical option for treatment- resistant mTLE, particularly among patients with MTS. Remission of presurgical mood and anxiety disorders can also result from LITT. |
| *Kim et al., 2022* | USA | 2014-2019 | - mTLE patients underwent LITT | - Patients who were part of the SLA for TLE trial (Medtronic) | Postsurgical seizure outcome using ILAE scale. | Greater extent of ablation of ADC hyperintensity cluster was significantly associated with complete seizure freedom in mTLE patients who underwent LITT. The results suggest that ADC may potentially identify patient-specific pathological and epileptogenic areas in the mTL that are high yield for ablation so can enable clinicians to optimize patient selection process, pre-operative planning, and intra-operative post-ablation assessment to further improve seizure outcomes. |
| *Landazuri et al., 2020* | USA | 5 years | - DR-mTLE patients underwent LITT | - NA | **1ry:** Seizure outcome by Engel classifications. **2ry:** Adverse events, hospitalization, discharge head pain and quality of Life in Epilepsy questionnaire. | Surgical treatment with LITT for epileptic foci is a safe and effective treatment option for people with drug resistant epilepsy.The minimally invasive nature allows for short hospitalizations with minimal reported pain and discomfort. |
| *Le et al., 2018* | USA | 2014 - 2017 | - Patients with refractory mTLE underwent LITT | - Lesional epilepsy other than MTS - Incomplete ablation | **1ry:** Seizure outcomes using Engel classification.  **2ry:** Reduction in baseline seizure frequency and postablation complications. | Laser ablation is well tolerated and offers marked seizure reduction for the majority of patients. |
| *Mo et al., 2023* | China | 2015-2023 | - DR-mTLE patients Underwent LITT or lobectomy - Unilateral epileptogenic zone in the anteromedial temporal region | - Intracranial space-occupying lesions - Dual pathology - Incomplete data - Reoperation history | Postsurgical seizure outcome using ILAE scale and postoperative seizure control prognosis. | Minimally invasive MRgLITT is associated with memory preservation and seizure control, similar to traditional open surgery. MRgLITT is effective and safe for DR-mTLE and is relevant for future prospective randomized trials on dominant-side mTLE, providing practical implications for guiding neurosurgeons in the selection of surgical approaches. |
| *Niu et al., 2023* | China | Jun-Nov 2021 | - Between 6 months and 70 years old - DR-mTLE patients - Signed informed consent forms by the patients or their families | - Contraindications for the MRI - Severe coagulation disorders - Pregnancy or lactation | **1ry:** Seizure outcomes using Engel classification.  **2ry:** Postoperative adverse effects. | As a minimally invasive method for the ablation of DR epileptic lesions, MRgLITT has high safety, a low complication rate, and a good surgical effect. It may even be used as a partial replacement for craniotomy in the future. However, due to the limitations of this study, a larger, multicenter, prospective MRgLITT study with a longer follow-up period is still required to improve the findings. |
| *Petito et al., 2018* | USA | 2013 - 2015 | - ≥ 18 y.o - DR-mTLE patients - Follow-up > 6 months | - < 18 y.o - Pregnant - Follow-up < 6 months | Seizure outcomes using Engel classification. | Seizure-free outcome with SLA and resection in lesional temporal epilepsy was similar but with a shorter length of stay after SLA |
| *Sun et al., 2024* | USA | 2014-2020 | - Having simultaneous scalp EEG and iEEG recordings - Having at least one depth electrode targeting the amygdalohippocampal complex using an occipital approach - Follow-up > 3 years - Undergoing MRI scans to identify the presence of MTS | - Patients with significant structural lesions like; tumors, stroke, or vascular malformations Previous open brain surgery history | Seizure outcomes using Engel classification. | Patients with MTS exhibited favorable short-term and long-term surgical outcome after SLAH. A higher number of i-SCSs was significantly associated with MTS in patients with mTLE. The number of i-SCSs tended to be higher in patients with Engel I-II surgical outcomes. The association between i-SCSs, MTS, and surgical outcomes in mTLE patients undergoing SLAH has significant implications for understanding the underlying mechanisms and identifying potential therapeutic targets to enhance surgical outcomes. |
| *Tao et al., 2017* | USA | 2014 - 2017 | - DR-mTLE patients   underwent MRgLITT | - One patient diagnosed with both mTLE and psychogenic non-ep- ilepsy seizures. | **1ry:** Seizure outcomes using Engel classification  **2ry:** Verbal memory and confrontational naming | MRI-guided LITT is a safe and effective alternative to selective amygdalohippocampectomy and anterior temporal lobectomy for mTLE with MTS. |
| *Vakharia et al., 2018* | USA | 2012-2016 | - mTLE patients underwent SLAH | - N/A | **1ry:** Seizure outcomes using Engel classification.  **2ry:** Ablation volumes achieved. | Computer assissted planning offers a safer, potentially improved seizure-free outcomes following LiTT for MTLE compared to manually planned trajectories. |
| *Willie et al., 2014* | Georgia | 2011-2013 | - Focal unilateral seizure onsets within mesial temporal structures - Twelve SLAH patients provided informed written consents | - N/A | Technical and clinical outcomes of SLAH with MRI guidance | Efficacy appears to approach that of open resection, especially in patients with MTS. Such minimally invasive techniques may be more desirable to patients and result in increased utilization of epilepsy surgery among the large number of medically intractable epilepsy patients. |
| *Wu et al., 2019* | USA | 2011 - 2017 | - mTLE patients underwent SLAH | - Follow-up < 1 year - Inadequate imaging for analysis | **1ry:** Seizure outcomes using Engel classification.  **2ry:** Location and volume of ablation and complications. | Ablations should prioritize the amygdala, hippocampal head, parahippocampal gyrus, and rhinal cortices for seizure freedom, as posterior extension has diminishing returns. |
| *Yossofzai et al., 2022* | USA and Canada | 2011 - 2019 | - DR-mTLE children patients - Underwent MRgLiTT or open surgery - Follow-up > 1 year | - Corpus callosotomy, neurostimulation, multilobar or hemispheric surgery - Lesion with maximal dimension > 60mm | **1ry:** Seizure outcomes.  **2ry:** Complications and hospital stay. | Seizure outcome of MRgLITT at 1 year posttreatment was inferior to open surgery. |
| *Youngerman et al., 2018* | USA | 2013 - 2016 | - DR-mTLE patients underwent LITT | - N/A | **1ry:** Seizure outcomes using Engel classification.  **2ry:** Length of stay, MT ablation, side, additional ablation site. | similar rates of seizure freedom following SLAH in patients with MTS and SEEG-confirmed, non-MTS mTLE. |
| *Youngerman et al., 2023* | USA | 2012 - 2018 | - DR-mTLE patients underwent LITT | - Previous resection or ablation for epilepsy or if the ablation was primarily targeting a mesial temporal lesion such as a tumour or cavernous malformation rather than the amygdalo-hippocampal complex and adjacent mesial cortex | Seizure outcomes using Engel classification. | MRgLITT is a viable treatment with durable outcomes for patients with drug resistant mTLE evaluated at a comprehensive epilepsy center. Although seizure freedom rates were lower than reported with ATL, this series represents the early experience of each center and a heterogeneous cohort. ATL remains a safe and effective treatment for well selected patients who fail MRgLITT. |

mTLE: mesial temporal lobe epilepsy; LITT: laser interstitial thermal therapy; MTS: mesial temporal sclerosis; MRgLITT: magnetic resonance guided laser interstitial thermal therapy; iECoG: intraoperative electrocorticography; SLAH: stereotactic laser amygdalohippocampotomy; ATL: anterior temporal lobectomy; ADC: apparent diffusion coefficient; DR: drug resistant; i-SCSs: subclinical seizures detected on iEEG; MRI: magnetic resonance imaging; SLA: stereotactic laser ablation; TLE: temporal lobe epilepsy; iEEG: intraoperative electroencephalogram; SEEG: stereoelectroencephalogram.
