# Supplementary material for: Efficacy and safety of laser interstitial thermal therapy versus radiofrequency ablation and stereotactic radiosurgery in the treatment of intractable mesial temporal lobe epilepsy: a systematic review and meta-analysis
Source: Neurosurg Rev. 2025 Jan 21;48(1):71. doi: 10.1007/s10143-025-03215-8 (PMC11750889; doi:10.1007/s10143-025-03215-8)
Supplement: Supplementary file 5 — Supplementary Material 5 [file 10143_2025_3215_MOESM5_ESM.docx]

**Laser interstitial thermal therapy versus radiofrequency ablation and stereotactic radiosurgery in the treatment of intractable mesial temporal lobe epilepsy: A systematic review and meta-analysis**

Youstina Mohsen^1^, Khalid Sarhan^2^, Ibrahim Saleh Alawadi^1^, Reem Reda Elmahdi^1^, Yasmeena Abdelall Kozaa^1^, Menna A. Gomaa^1^, Ibrahim Serag^2^, Mostafa Shahein^3^

^1^Mansoura Manchester Program for Medical Education (MMPME), Faculty of Medicine, Mansoura University, Mansoura, Egypt

^2^Faculty of Medicine, Mansoura University, Mansoura, Egypt

^3^Department of neurosurgery, Faculty of Medicine, Mansoura University, Egypt

CORRESPONDING AUTHOR:

Youstina Mohsen

Email: [youstinamohsen1@std.mans.edu.eg](mailto:youstinamohsen1@std.mans.edu.eg), [youstinamosensamir@gmail.com](mailto:youstinamosensamir@gmail.com)

ORCID: 0000-0002-5949-1794

Submitted to Neurosurgical Review journal

**
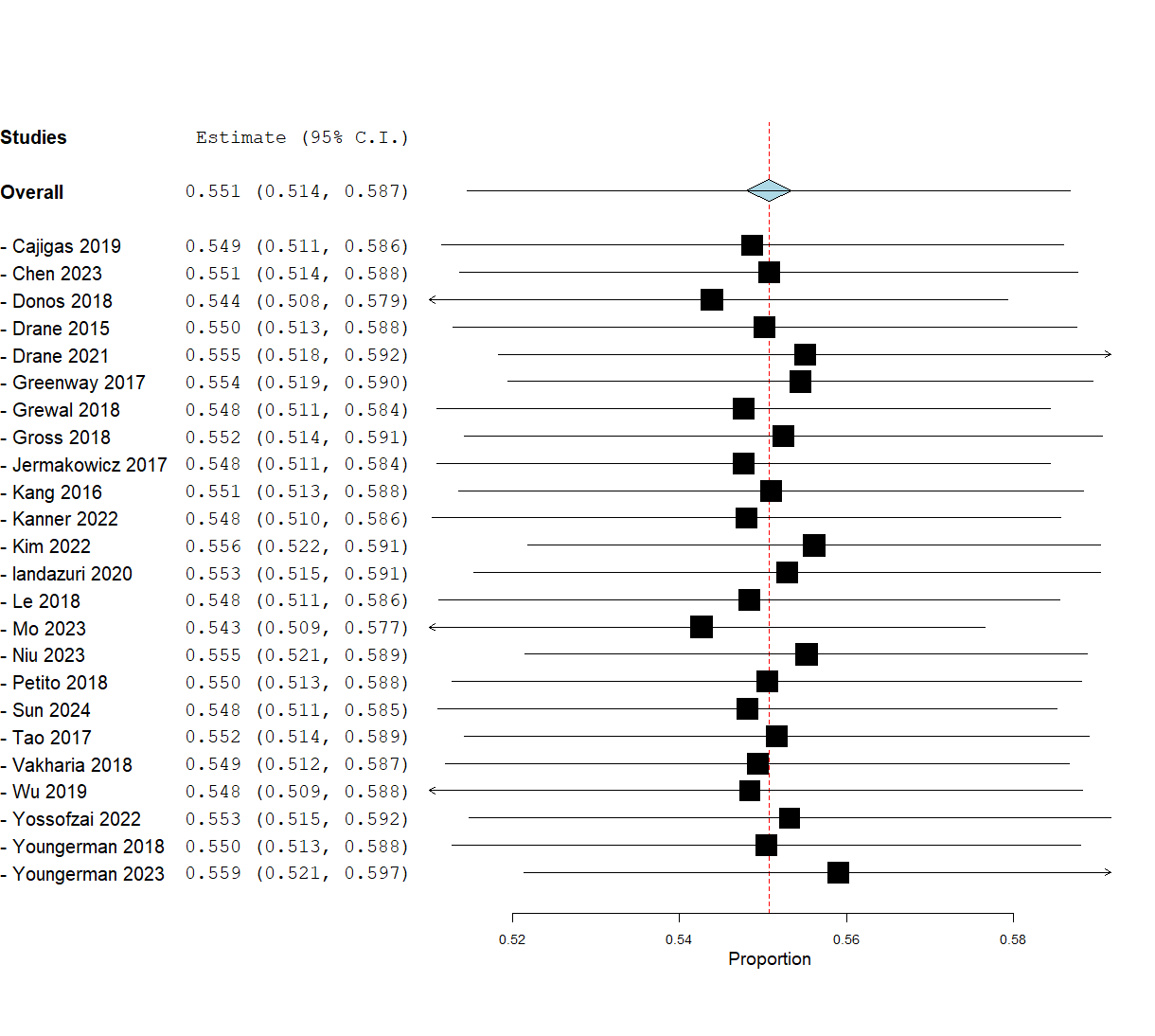
Figures for sensitivity analysis**

Figure 1: Engel I outcome for LITT


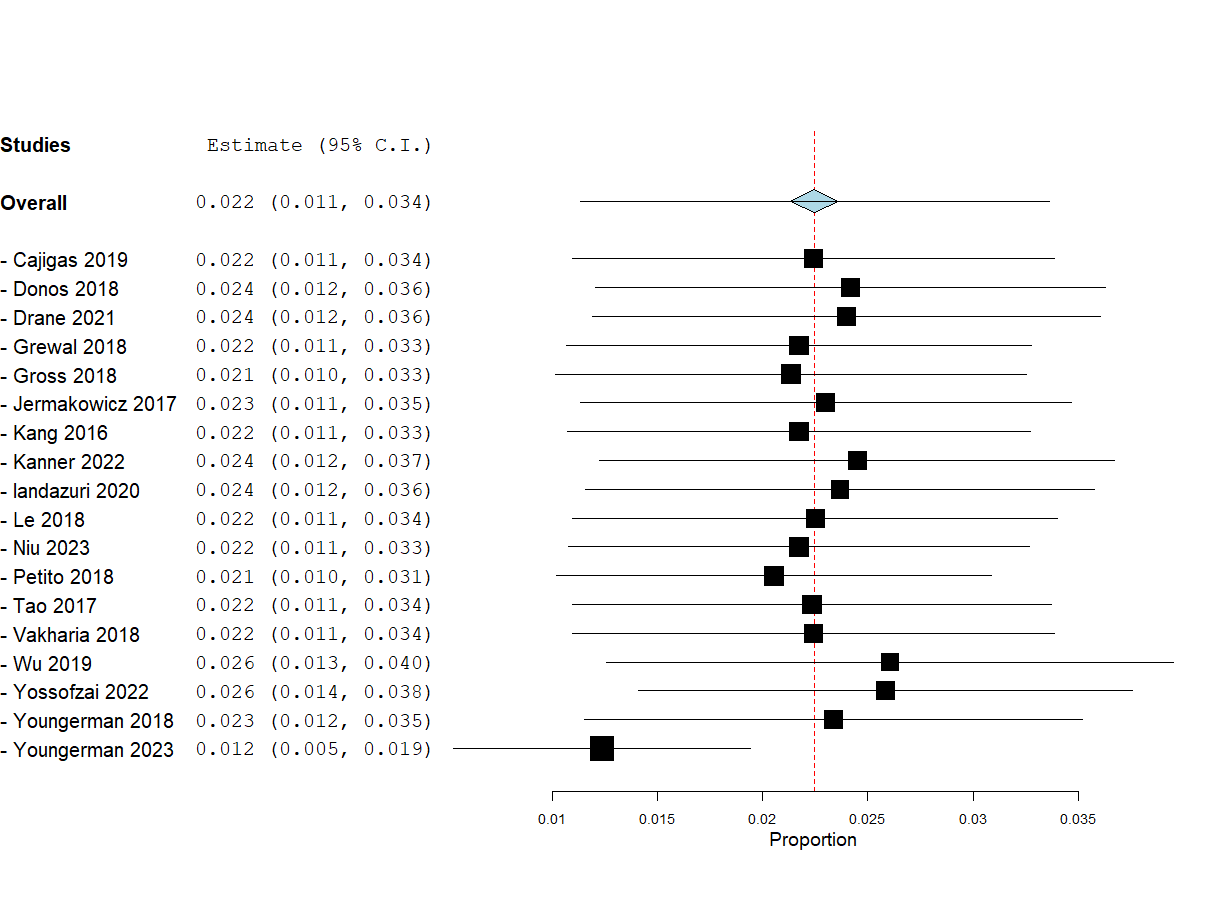


Figure 2: Complications rate for LITT


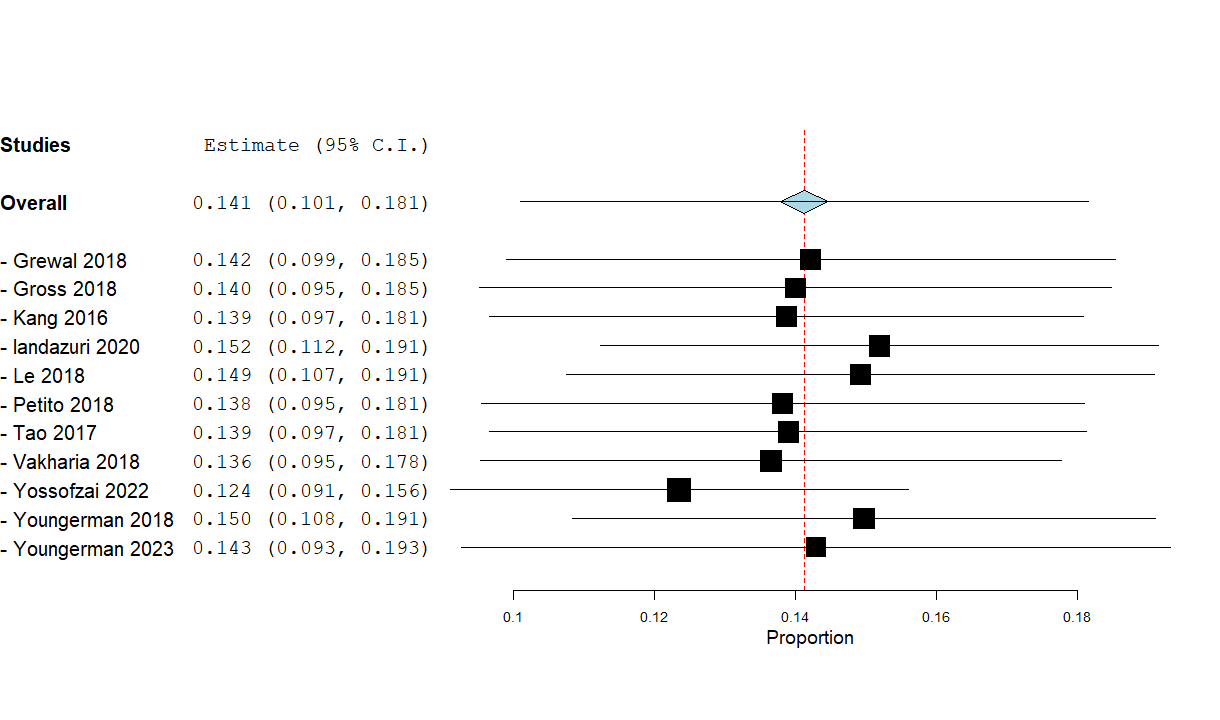


Figure 3: Reoperation rate for LITT


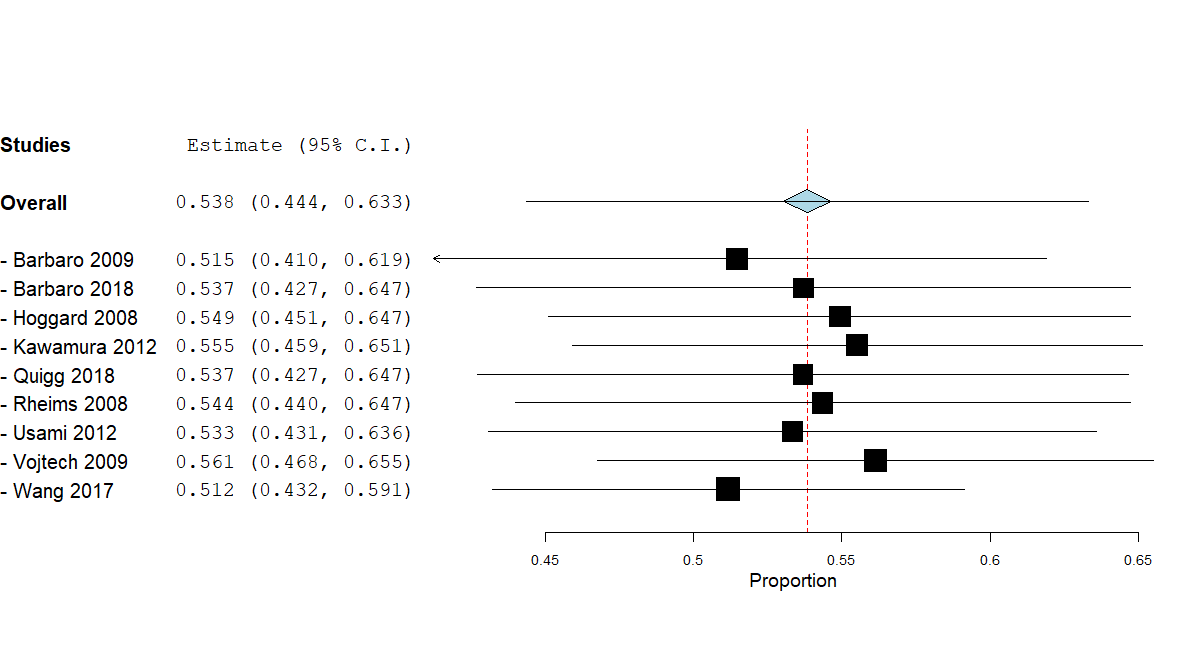


Figure 4: Engel I outcome for SRS


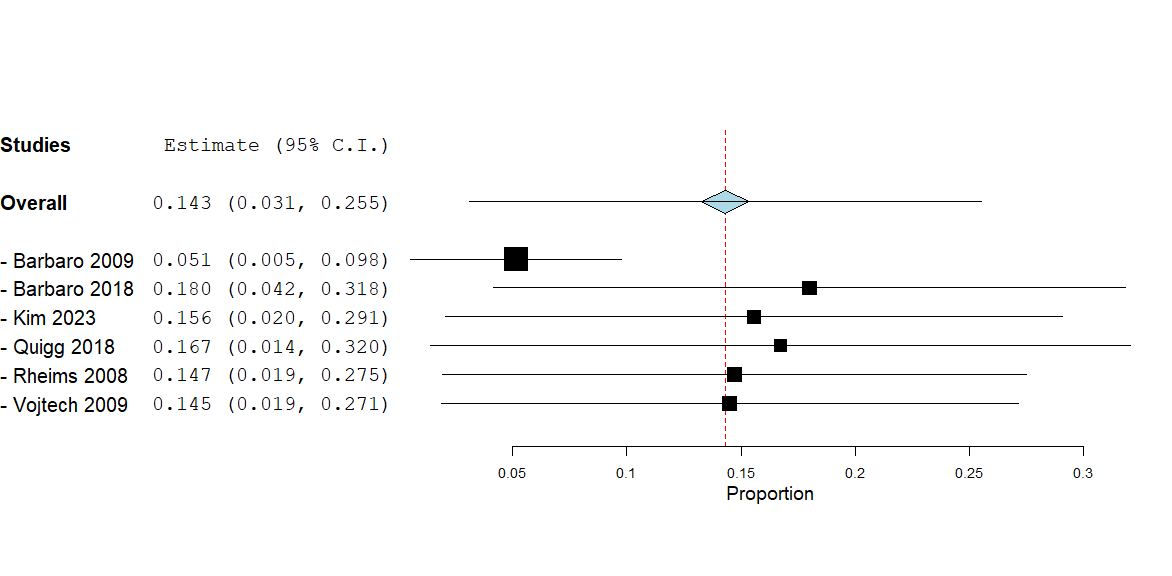


Figure 5: Complications rate for SRS


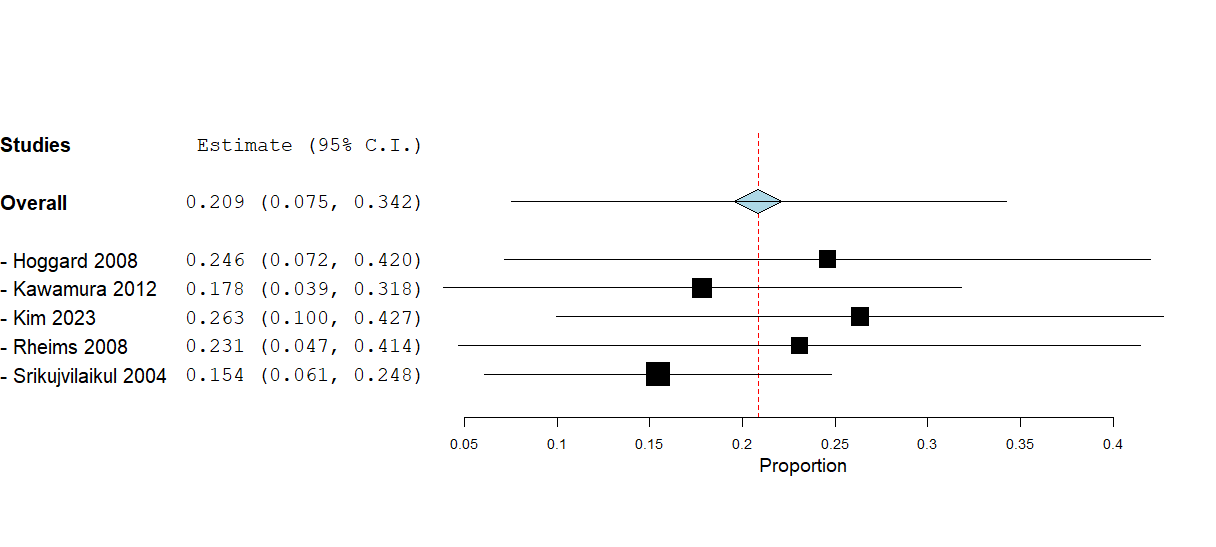


Figure 6: Reoperation rate for SRS


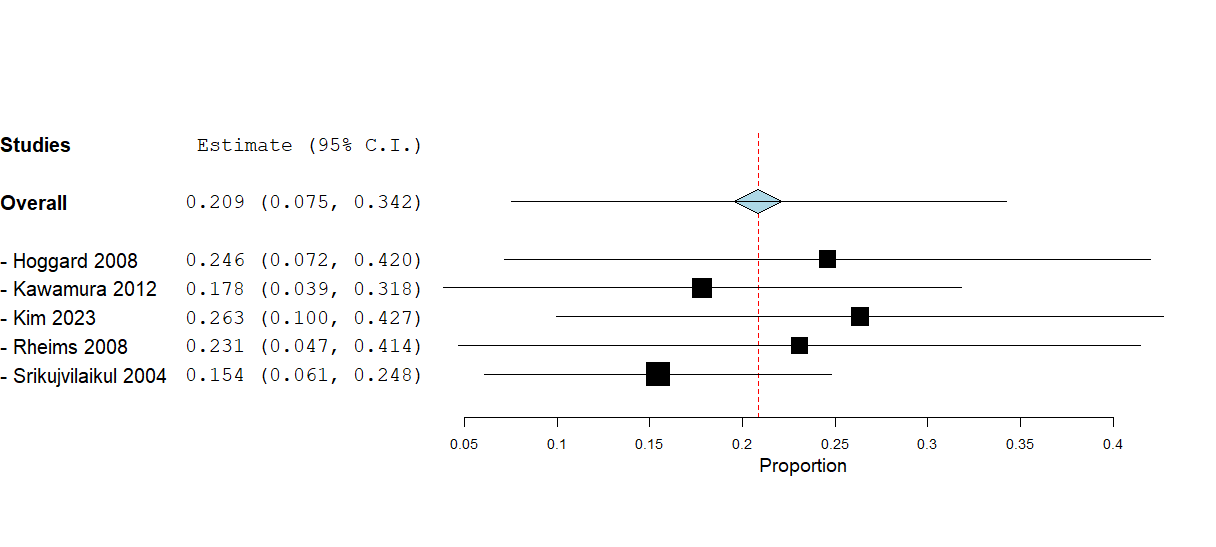


Figure 7: Engel I outcome for RFA


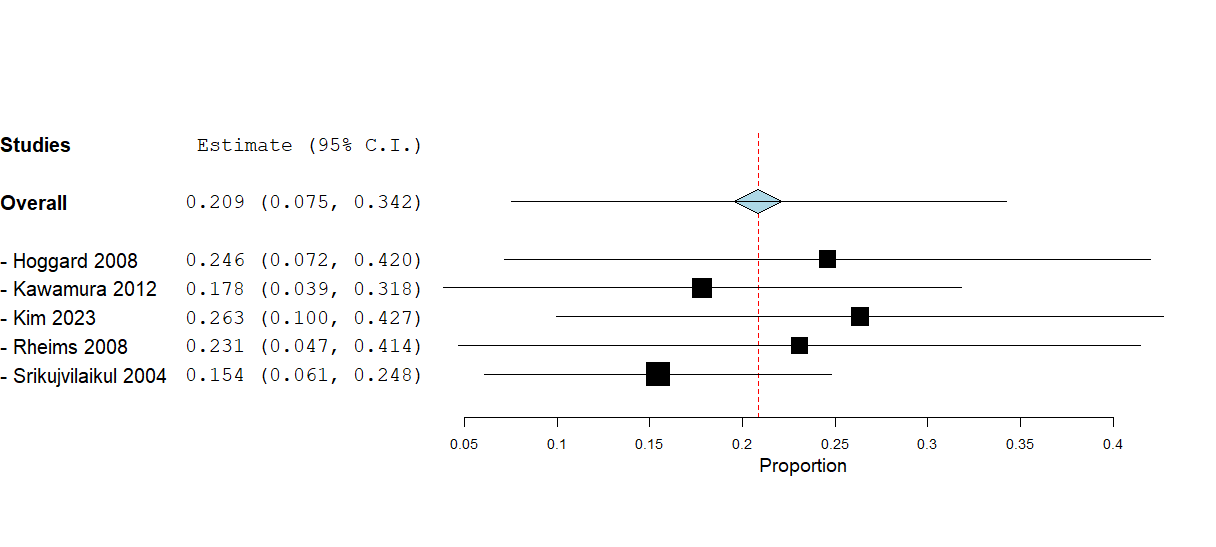


Figure 8: Complications rate for RFA


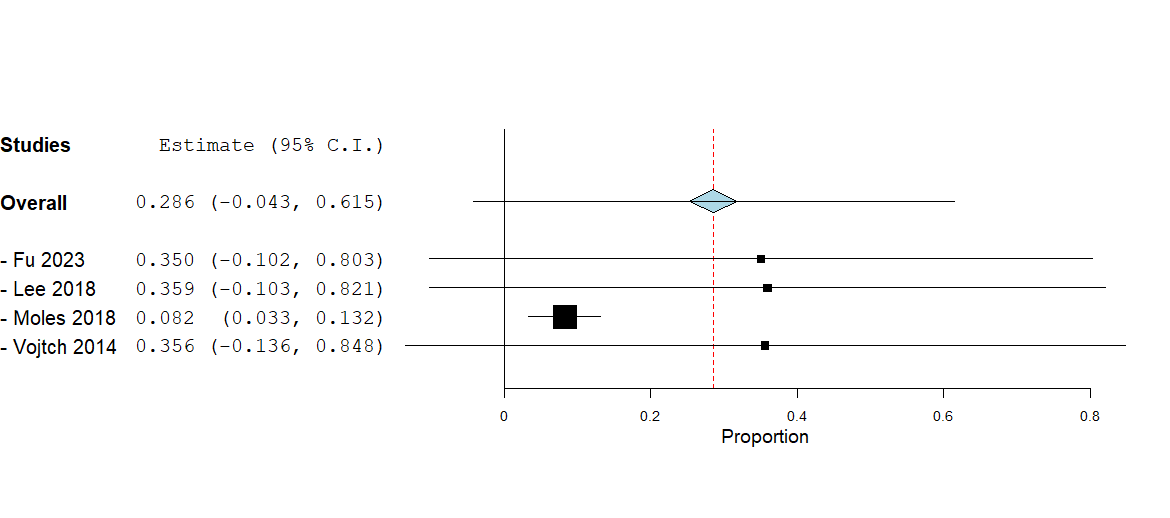


Figure 9: Reoperation rate for RFA
