# Supplementary material for: Efficacy and safety of laser interstitial thermal therapy versus radiofrequency ablation and stereotactic radiosurgery in the treatment of intractable mesial temporal lobe epilepsy: a systematic review and meta-analysis
Source: Neurosurg Rev. 2025 Jan 21;48(1):71. doi: 10.1007/s10143-025-03215-8 (PMC11750889; doi:10.1007/s10143-025-03215-8)
Supplement: Supplementary file 6 — Supplementary Material 6 [file 10143_2025_3215_MOESM6_ESM.docx]

**Laser interstitial thermal therapy versus radiofrequency ablation and stereotactic radiosurgery in the treatment of intractable mesial temporal lobe epilepsy: A systematic review and meta-analysis**

Youstina Mohsen^1^, Khalid Sarhan^2^, Ibrahim Saleh Alawadi^1^, Reem Reda Elmahdi^1^, Yasmeena Abdelall Kozaa^1^, Menna A. Gomaa^1^, Ibrahim Serag^2^, Mostafa Shahein^3^

^1^Mansoura Manchester Program for Medical Education (MMPME), Faculty of Medicine, Mansoura University, Mansoura, Egypt

^2^Faculty of Medicine, Mansoura University, Mansoura, Egypt

^3^Department of neurosurgery, Faculty of Medicine, Mansoura University, Egypt

CORRESPONDING AUTHOR:

Youstina Mohsen

Email: [youstinamohsen1@std.mans.edu.eg](mailto:youstinamohsen1@std.mans.edu.eg), [youstinamosensamir@gmail.com](mailto:youstinamosensamir@gmail.com)

ORCID: 0000-0002-5949-1794

Submitted to Neurosurgical Review journal


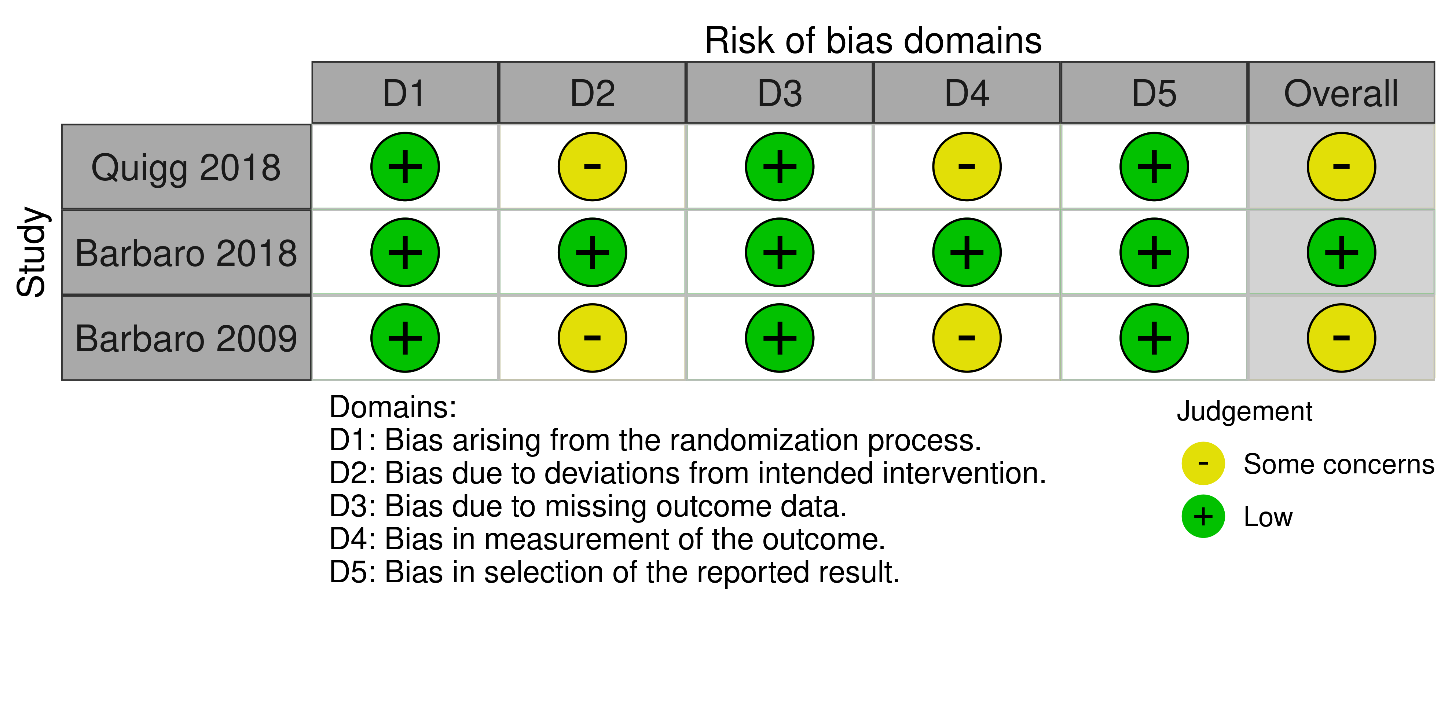


Figure 1: quality assessment of RCTs using ROB2 tool.

| Study ID | The Joanna Briggs Institute (JBI) Critical Appraisal checklist for Case Reports | | | | | | | | Quality  (Total Quality Score) |  |
| --- | --- | --- | --- | --- | --- | --- | --- | --- | --- | --- |
|  | Were  the patient's demographic  characteristics  clearly described? | Was the patient’s history clearly described and presented as a timeline? | Was the Current clinical condition of the patient on presentation clearly described? | Were diagnostic tests or assess ment methods and the results clearly described? | Was the intervent ion(s) or treatment procedure(s)clearly described? | Was the post- intervention clinical condition clearly described? | Were Adverse events(harms) or unanticipated events identified and described? | Does the Case report  Provide takeaway lessons? |  | Final Appraisal |
| Cajigas 2019 | yes | yes | yes | No | yes | yes | yes | yes | 7 | High quality |
| Donos 2018 | yes | yes | No | yes | yes | No | No | yes | 5 | Moderate quality |
| Jermakowicz 2017 | yes | yes | yes | yes | yes | yes | No | yes | 7 | High quality |
| Fan 2019 | yes | yes | yes | No | No | yes | No | yes | 5 | Moderate quality |
| Hoggard 2008 | yes | yes | yes | yes | yes | yes | No | yes | 7 | High quality |
| Usami 2012 | yes | yes | yes | No | No | yes | No | yes | 5 | Moderate quality |

Table 1: quality assessment of cohort studies using JBI tool.

Figure 2: quality assessment of non-randomized studies using MINORS tool.
